# Pulsed electromagnetic stimulation promotes neuronal maturation by up-regulating cholesterol biosynthesis

**DOI:** 10.1186/s13287-025-04469-1

**Published:** 2025-07-26

**Authors:** Ping Chen, Jingyi Li, Vsevolod Telezhkin, Yu Gu, Min Tao, Liping Guo, Simin Song, Rihe Dong, Xianyang Luo, Yan Wang, Qian Liu, Weiming Tian, Weihua Meng, Wei Hong, Bing Song

**Affiliations:** 1 Faculty of Biomedical Engineering, Shenzhen University of Advanced Technology, Shenzhen, China; 2 Nottingham Ningbo China Beacons of Excellence Research and Innovation Institute, University of Nottingham Ningbo China, Ningbo, China; 3 Shenzhen Key Laboratory of Neuroimmunomodulation for Neurological Diseases, Shenzhen-Hong Kong Institute of Brain Science, Shenzhen Institutes of Advanced Technology, Chinese Academy of Sciences, Shenzhen, 518055 China; 4 Translational Medical Research and Development Center, Institute of Biomedical and Health Engineering, Shenzhen Institutes of Advanced Technology, Chinese Academy of Sciences, Shenzhen, China; 5 School of Dental Sciences, Faculty of Medical Sciences, Newcastle University, Newcastle Upon Tyne, NE2 4BW UK; 6 Morrello Clinic, Neuro Rehabilitation and Neuro Physiotherapy, Newport, NP18 2LH UK; 7 School of Life Science and Technology, Harbin Institute of Technology Harbin, Harbin, China; 8 Division of Population Health and Genomics, Ninewells Hospital and Medical School, University of Dundee, Dundee, UK; 9 Center for Public Health, Faculty of Medicine, Health and Life Sciences, School of Medicine, Dentistry and Biomedical Sciences, Queen’s University Belfast, Belfast, UK; 10 Key Laboratory of Biomedical Imaging Science and System, Chinese Academy of Sciences, and State Key Laboratory of Biomedical Imaging Science and System, Shenzhen, China; 11 School of Dentistry, Cardiff University, Heath Park, Cardiff, CF14 4XY, UK

**Keywords:** Induced pluripotent stem cells, Neuronal differentiation, Pulsed electromagnetic fields, Synaptic marker, Cholesterol biosynthesis

## Abstract

**Background:**

Stem cell therapies have emerged as transformative therapeutic strategies for neurological disorders. However, neurons derived from transplanted stem cells often exhibit low survival rates and remain in an immature state. While pulsed electromagnetic fields (PEMF) may enhance neuronal differentiation, the extent of this effect and its molecular mechanisms remain poorly characterized.

**Method:**

Human induced pluripotent stem cells (iPSCs) induced cortical neurons received daily PEMF stimulation (1 mT, 15 Hz, 3.75 ms pulse duration) for 7 days during differentiation. Neuronal differentiation and synaptic maturation were assessed using immunocytochemistry, qPCR, western blotting, and live-cell imaging to evaluate neurite outgrowth. Functional maturation was analyzed through calcium imaging and patch-clamp electrophysiology. Transcriptomic profiling identified key pathways involved in PEMF-modulated neuronal maturation, with the role of FDFT1-mediated cholesterol biosynthesis mechanistically validated through pharmacological inhibition and genetic knockdown.

**Result:**

PEMF accelerated early-stage neuronal differentiation without altering neurite outgrowth and enhanced synaptic maturation after sustained stimulation. PEMF-treated neurons displayed heightened spontaneous calcium signaling and improved functional maturation, including enhanced excitability, action potential kinetics, and voltage-gated ion channel activity. Transcriptomics revealed significant upregulation of cholesterol biosynthesis pathways, with FDFT1 (squalene synthase) as a central regulator. Pharmacological inhibition or genetic knockdown of *FDFT1* abolished PEMF-induced neuronal differentiation and synaptic maturation.

**Conclusion:**

PEMF accelerates early-stage differentiation of human cortical neurons and enhances synaptic maturation following sustained stimulation. These effects are mechanistically linked to the activation of FDFT1-mediated cholesterol biosynthesis. This non-invasive PEMF stimulation approach represents a promising strategy to optimize stem cell-based therapies for neurological disorders.

**Supplementary Information:**

The online version contains supplementary material available at 10.1186/s13287-025-04469-1.

## Introduction

The escalating global prevalence of neurological disorders—including stroke, spinal cord injuries, and neurodegenerative diseases—has imposed a profound socioeconomic burden over recent decades [1]. Stem cell therapy represents a transformative approach in regenerative medicine, offering the potential to repair, replace or regenerate damaged neurons. Stem cells are well known for their potent self-renewal and differentiation capabilities, and have been extensively studied in the treatment of degenerative diseases, injuries and genetic disorders [2–4]. Notably, induced pluripotent stem cells (iPSCs), reprogrammed from somatic cells (e.g., dermal fibroblasts or peripheral blood mononuclear cells) through ectopic expression of four core transcription factors [5]. These iPSCs possess similar pluripotency to embryonic stem cells (ESCs), allowing them to differentiate into any cell type with appropriate manipulations [5, 6].

To date, two primary methods are widely used to generate functional neurons from human iPSCs. The first method employs a stepwise approach that utilizes a combination of small molecules and growth factors, recapitulating developmental stages from neural progenitors to mature neurons [7–10]. The second utilizes forced expression of neurogenic transcription factors (e.g., Ascl1, NeuroD1) to directly reprogram iPSCs or somatic cells into induced neurons (iNs), achieving accelerated maturation compared to growth factor-driven approaches [11–15]. For instance, Neurogenin 2 (Ngn2) is a transcription factor that plays a crucial role in regulating gene expressions involved in neuronal development. Human neurons engineered from iPSCs through Ngn2 overexpression have been widely used in modeling neuronal development and neurological diseases [16–20].

However, accumulating evidence revealed that Ngn2-induced neurons showed functional immaturity in Ngn2-induced neurons, characterized by attenuated action potentials, limited spontaneous synaptic activity and delayed network synchronization—persisting even after 21 days of differentiation. In contrast, neurons generated by a more natural but slow path of neurogenesis, the embryoid body (EB)-based dual SMAD inhibition procedure, can reach a more advanced grade of neuronal maturity than Ngn2-derived neurons. In fact, the timescale required for neuronal maturation and the functions of resulted neuronal-like cells are likely attributed to their distinct gene expression patterns [21, 22]. Additionally, transplanted human iPSC-derived neurons displayed low survival rates, even when supplemented with growth factors that may promote their neuralization [23–25]. Thus, the imperfect procedures of neuronal differentiation and the consequently generated immature neurons are not able to meet the requirements for stem cell transplantation and rehabilitation.

Pulsed electromagnetic fields (PEMF) stimulation has garnered increasing attention as a non-invasive physical modality for cell differentiation in tissue engineering and regenerative medicine and has been approved for promoting bone repair [26, 27]. PEMF stimulation augmented alkaline phosphatase (ALP) activity and calcium deposition and accelerated the expression of various osteogenic markers to promote osteogenic differentiation of mesenchymal stem cells [28]. PEMF stimulation was shown to effectively promote the differentiation of skeletal muscle fibers by activating mitochondrial-based survival adaptations [29]. This technique is also being used in the regeneration of damaged neural tissues by enhancing the proliferation and neurodifferentiation of the neural stem cells (NSCs), as well as increasing the expression of neurotrophic factors, such as brain-derived neurotrophic factor (BDNF) [30, 31]. Specifically, PEMF at a frequency of 15 Hz and an intensity of 2 mT can induce significant calcium (Ca^2+^) oscillations and produce robust Ca^2+^ spikes [32]. Spontaneous Ca^2+^ oscillations, spikes and influx are crucial in neurite outgrowth and branching, enabling functional synapse formation and stabilization [33–35]. However, there is no clear demonstration of the dominant mechanism involved in the regulation of neuronal differentiation and activity by PEMF stimulation.

In this study, we took advantage of well-characterized Ngn2-induced cortical neurons and an array of cellular and electrophysiological assays, to examine the function and mechanism of PEMF in promoting neuronal differentiation. We found that PEMF stimulation promoted the early-stage neuronal differentiation and accelerated the expression of synaptic proteins independent of neurite outgrowth following sustained treatment. PEMF enhanced the spontaneous calcium activity and improved the excitability action potential of iPSC-derived neurons (iNs) by increasing transmembrane Na^+^ voltage-gated conductance. Transcriptomic analysis revealed that cholesterol biogenesis pathway was up-regulated upon PEMF stimulation and a key gene, *FDFT1* that encodes squalene synthase, was the most significantly modulated. We found that pharmaceutically blockage of *FDFT1* activity impaired the formation of neuronal structural network and reduced the expression of synaptic proteins in the absence of PEMF, and abolished PEMF-promoted neuronal maturation. Genetic knockdown of *FDFT1* abolished the enhancement of synaptic protein expression following PEMF treatment. These data suggest that cholesterol biogenesis pathway plays a critical role in PEMF boosted-neuronal maturation and that such non-invasive stimulation techniques might be useful in the transplantation therapies of neurological diseases and regenerative medicine.

## Materials and methods

### Human induced pluripotent stem cell neuronal differentiation and in vitro culturing

The DYR0100 human iPSCs line was obtained from the Stem Cell Bank at the Chinese Academy of Sciences and maintained in mTeSR media (Cat# 85,850; STEMCELL Technologies, Vancouver BC, Canada). Human iPSC-derived neurons (iNs) were prepared according to a previously described procedure with minor modifications in which forced expression of Ngn2 was performed to turn on the glutamatergic neuron differentiation [20, 36]. Lentiviruses were prepared by Taitool Bioscience (Shanghai, China) with ultrapure titers and used at the following multiplicity of infection (MOI): Tet-O-Ngn2-puro (MOI = 5), FUdeltaGW-rtTA (MOI = 3). Neuronal differentiation was induced by doxycycline (Cat# D9891-5g; Sigma-Aldrich, St. Louis, MO, USA) at a final concentration of 2 μg/ml. To switch on Ngn2 expression, doxycycline was added to the neuronal differentiation media at a concentration of 2 μg/mL on day 1 of lentiviral infection. On the next day, puromycin was added to the neuronal differentiation media at a concentration of 10 μg/mL and this concentration was maintained thereafter. By day 4 post-lentiviral infection, cells were frozen down or plated at a density of 12,500 cells/cm^2^ on the 96-well plate pre-coated with Matrigel (Cat# 354,277; Corning, NY, USA) and poly-L-ornithine (Cat# P4957-50ML; Sigma-Aldrich, St. Louis, MO, USA). Thereafter, cells were maintained in media consisting of Neurobasal medium (Cat# 21,103,049; Gibco, NY, USA), Glutamax (100 ×) (Cat# 35,050–061; Gibco, NY, USA), 20% Dextrose, MEM non-essential amino acids (Cat# 11,140–050; Gibco, NY, USA), and B27 (50 ×) (Cat# 17,504–044; Gibco, NY, USA), along with brain-derived neurotrophic factor (BDNF) (Cat#4,500,250; Peprotech, Rocky Hill, NJ, USA), ciliary neurotrophic factor (CNTF) (Cat#4,501,350; Peprotech, Rocky Hill, NJ, USA), and glial cell-derived neurotrophic factor (GDNF) (Cat#4,501,050; Peprotech, Rocky Hill, NJ, USA) at a concentration of 10 ng/mL each.

### Pulsed electromagnetic field (PEMF) stimulation

To meet the basic requirements of the experiment, a custom-made PEMF generator was constructed using a Helmholtz coil and a self-designed driving system. This system provided a uniform pulsed electromagnetic field in a central region measuring 120 mm × 80 mm. At 24 h after iNs plating, the 96-well plate was transferred into the central region and exposed to PEMF for 2 h per day for 7 days. The PEMF signal was characterized by an intensity of 1 mT, a pulse duration of 3.75 ms, and a frequency of 15 Hz.

### Western blot

Cells were harvested and washed twice with ice-cold phosphate-buffered saline (PBS), followed by lysis in RIPA buffer (50 mM Tris–HCl, pH 7.4, 150 mM NaCl, 1% NP-40, 0.5% sodium deoxycholate, 0.1% SDS) supplemented with Phenylmethylsulfonylfluoride (PMSF) (Cat# 36,978; Thermo Fisher Scientific, Waltham, MA, USA). 10% SDS-PAG gels resolved the lysate samples and subsequently transferred to nitrocellulose membranes (Cat# 10,600,001; cytiva, Marlborough, MA, USA) using a wet transfer system at 160 mA for 90 min on ice. The membranes were incubated with primary antibodies targeting at: synaptophysin (1:1000, Cat#837,104; biolegend, San Diego, CA, USA) and GAPDH (1:5000, Cat#10,494–1-AP; proteintech, Rosemont, IL, USA) 4℃ overnight after 1 h 1% BSA blocking of the membranes. After washing six times with tris-buffered saline with Tween 20 (5 min each), membranes were incubated with secondary antibodies (1:20,000, Cat# 5257S; Cell Signaling Technology, Danvers, MA, USA, 1:20,000, Cat# 5366S; Cell Signaling Technology, Danvers, MA, USA) in 1% BSA in TBST for 1 h at room temperature. The blots were visualized by Odyssey Infrared Imaging System (LI-COR Biosciences, Lincoln, NE, USA). The intensity of the bands was assessed using image J software throughout. The expression of synaptophysin was normalized to the GAPDH.

### Calcium imaging and data analysis

Lentivirus of Ngn2 (TeT-O-NGN2-puro), rtTA (Fudelta GW-rtTA) and jGCaMP7 (UbiC-jGCaMP7s) were obtained from Taitool Bioscience (Shanghai, China). A combination of the three viruses were added into iPSC and doxycycline was added to induce the neuronal differentiation of iPSC. The expression of calcium indicator jGCaMP7s could be detected after the lentivirus infection of iPSC and variance in the glowing intensity of the green fluorescent protein (GFP) displayed the calcium activity in the iNs. Calcium imaging of iNs was performed by using Olympus IXplore SpinSR10 Super-Resolution Imaging System (Olympus, Tokyo, Japan). iNs were plated in a 96-well plate (Cat# 655,090; greiner, Frickenhausen, Germany) and placed in the microscope chamber maintained with 37 °C, 5% CO_2_, and 95% humidity. A 488 nm laser was employed for excitation, and images were captured at a frequency of 5 Hz, with each sample consisting of three replicate wells. The background was subtracted to analyze the fluorescence intensity (F). The baseline fluorescence was denoted as F_0_, and the change in the fluorescence (_Δ_F) was calculated as _Δ_F = F−F_0_. The ratio ΔF/F served as an index indicating calcium fluctuations'dynamics. Calcium wave analysis was conducted by performing power spectrum analysis, followed by calculations of power spectrum and power spectral density (PSD) using a generic function in Python.

### RNA extraction and quantitative real-time PCR

Total RNA was extracted using TRIzol reagent (Cat# T9424; Sigma-Aldrich, St. Louis, MO, USA) according to the manufacturer’s instructions. The total yield of RNA was measured by a NanoDrop spectrophotometer. For reverse transcription, 1 µg of total RNA was converted to cDNA using the Hifair® III 1 st Strand cDNA Synthesis SuperMix for quantitative real-time PCR (qPCR) (Cat# 11141ES10; Yeasen biotechnology, Beijing, China). QPCR was performed using the SYBR Green PCR Master Mix (Cat# 11184ES08; Yeasen biotechnology, Beijing, China) on the QuantStudio 7 Real-Time PCR System (Applied Biosystems, Foster City, CA, USA). The sequences of primers were shown in the Table 1. The qPCR cycling conditions were as follows: initial denaturation at 95 °C for 2 min, followed by 40 cycles of 95 °C for 10 s and 60 °C for 30 s. The mRNA expression of GAPDH was set as internal control and relative to a control sample (untreated cells). The relative quantification in gene expression was determined using the 2^−ΔΔCt^ method.

**Table 1:** Primers used in this study

Primer	Sequence 5′–3′	Accession number
FDFT1-F	CTGCACCACATCCCAGATGT	NM_001287756.2
FDFT1-R	CCGAATCTTCACTGCCCCTT	
LDLR-F	AATGCTTGGACAACAACGGC	NM_001195803.2
LDLR-R	CGGGATCCTGACACTCATCG	
SREBP2-F	CCTGGAAGTGACAGAGAGCC	NM_004599.4
SREBP2-R	TCGCAATGGCAGAAGGAACT	
HMGCR-F	GGTGATGGGAGCTTGTTGTG	NM_001364187.1
HMGCR-R	GCACCTCCACCAAGACCTA	
SYP-F	TCGGCTTTGTGAAGGTGCTGCA	NM_003179.3
SYP-R	TCACTCTCGGTCTTGTTGGCAC	
SYN1-F	CGATGCCAAATATGACGTGCGTG	NM_133499.2
SYN1-R	AGCATCGCAGAGCCAGTATTGG	
PSD95-F	TCCACTCTGACAGTGAGACCGA	NM_001321075.3
PSD95-R	CGTCACTGTCTCGTAGCTCAGA	
OCT4-F	GTGGTCCGAGTGTGGTTCTGTAAC	NM_203289.6
OCT4-R	CCCAGCAGCCTCAAAATCCTCTC	
SOX2-F	CAGCATGTCCTACTCGCAGCAG	NM_003106.4
SOX2-R	CTGGAGTGGGAGGAAGAGGTAA	
Nestin-F	TCAAGATGTCCCTCAGCCTGGA	NM_006617.2
Nestin-R	AAGCTGAGGGAAGTCTTGGAGC	
GAPDH-F	GTGGACCTGACCTGCCGTCTAG	NM_001289746.2
GAPDH-R	GAGTGGGTGTCGCTGTTGAAGTC	

### RNA sequencing

The RNA was extracted from iNs with and without PEMF stimulation, using the RNeasy Micro Kit (Cat# 74,004; QIAGEN, Hilden, Germany) following the manufacturer’s instructions. After extraction, the RNA was assessed for quality and quantity using the Agilent 2100 Bioanalyzer (Agilent, CA, USA). Sample libraries were prepared with the Optimal Dual-mode mRNA Library Prep Kit (BGI, Shenzhen, China) and then amplified using phi29 and rolling circle amplification to create DNA nanoballs. Each DNB contained over 300 copies of the initial single-stranded circularized library molecule. The DNBs were loaded onto a patterned nanoarray, and paired-end 100/150 base reads were generated on the G400/T7/T10 platform (BGI, Shenzhen, China). The raw data were filtered using SOAPnuke (v1.6.5) and then expressed in terms of transcripts per million. Differential gene expression analysis was conducted using DESeq2 (v1.40.2), with an adjusted *P* value threshold of < 0.05. To explore the biological significance of the differentially expressed genes (DEGs), Gene Ontology (GO) and Kyoto Encyclopedia of Genes and Genomes (KEGG) pathway enrichment analysis were performed using the TermFinder package and the phyper function in R software, respectively. Significantly enriched GO terms and KEGG pathways were determined based on adjusted *P* values < 0.05.

### Live cell imaging and data analysis

On day 4, neurons were plated in a 96-well plate (Cat# 655,090; greiner, Frickenhausen, Germany) and placed in an IncuCyte S3 live-cell imaging instrument (Essen Bioscience, Ann Arbor, MI) for continuous imaging. Four fields per well were imaged every 12 h for 7 days. MF stimulation was conducted during the 12-h intervals when no images were captured. The entire set of images was analyzed using IncuCyte 2021 A Software (Essen Bioscience, Ann Arbor, MI) [37]. The analysis job, Neural Track, was utilized to automatically define neurite processes and cell bodies based on phase contrast images. Typical settings included: Segmentation Mode—Brightness; Segmentation Adjustment—1.2; Cell Body Cluster Filter—minimum 300 μm^2^; Neurite Filtering—Best; Neurite Sensitivity—0.65; Neurite Width—1 μm. Total neurite length was calculated per area over time.

### Immunocytochemistry, confocal microscopy and quantification

iNs were exposed to PEMF stimulation for 7 days. After PEMF stimulation, iNs were fixed in cold 4% formaldehyde in PBS buffer for 15 min and then quenched by 0.3M Glycine for 10 min. Then iNs were rinsed three times with PBS, permeabilized by 0.25% triton in PBS for 5 min at room temperature. After blocking with 3% BSA in PBS, the primary rabbit antibody for synaptophysin (1:1000, Cat# 67,864–1-Ig; Proteintech, Rosemont, IL, USA), mouse monoclonal antibody for NeuN (1:400, Cat#ab104224; Abcam, Cambridge, UK), mouse monoclonal antibody for PSD95 (1:200, Cat# ab192757; Abcam, Cambridge, UK) and chicken monoclonal antibody for β3-tubulin (1:1000, Cat# ab41489,; Abcam, Cambridge, UK) were applied to the iNs and incubated at 4 °C overnight (~ 12 h). Then iNs were rinsed with 0.05% tween-20 in PBS (PBST) for three times, and the secondary fluorescently labeled antibodies were applied and incubated at room temperature for 1 h. After rinsing three times with PBST, DAPI (Cat# C1002; Beyotime, Shanghai, China) was applied to DYR0100 iNs for labeling nuclei. Confocal microscopy was performed using Olympus Ixplore SpinSR10 Super-Resolution Imaging System (Olympus, Tokyo, Japan). Image J was used for fluorescent quantification.

### Electrophysiological recordings and data analysis

Voltage and current recordings were made using a conventional patch-clamp in the whole-cell configuration [38, 39]. The bath solution contained (in mM): 135 NaCl, 5 KCl, 1.2 MgCl_2_, 1.25 CaCl_2_, 10 D-glucose, 5 N-2-hydroxyethylpiperazine-N’−2-ethanesulfonic acid; pH was adjusted to 7.4 using 1 M NaOH. The pipette solution contained (in mM): 117 KCl, 10 NaCl, 11 N-2-hydroxyethylpiperazine-N’−2-ethanesulfonic acid (HEPES), 2 Na_2_-ATP, 2 Na-GTP, 1.2 Na_2_-phosphocreatine, 2 MgCl_2_, 1 CaCl_2_ and 11 ethylene–glycol-tetra-acetic acid; pH was adjusted to 7.2 with 1M KOH. All chemicals were purchased from Sigma-Alrich. All electrophysiological studies were performed using an Axopatch 200B amplifier and Digidata 1550B Data Acquisition System (Molecular Devices, LLC, 3860 N First Street, San Jose, CA 95134, USA) at a controlled room temperature. Recordings were digitized at 10 kHz and low-pass filtered at 5 kHz using an 8-pole Bessel filter and recorded using Clampex 11.1.

Resting membrane potential (V_m_) of DYR0100 human iNs was recorded in current-clamp mode. Neurons could be categorized into two separate groups of Quiet and Spontaneous. Once V_m_ and non-induced activity had been recorded, the current was injected to hyperpolarize V_m_ to ca. −80 mV before 1 s current injection steps were imposed (from −10 pA to + 190 pA) in order to induce action potentials. Neurons were coded according to the ability to generate induced action potential activity (iAP) that they demonstrated [40], defined as follows: (1) Quiet (no significant excursions from baseline during injection); (2) Attempting single (significant excursions from baseline that do not reach 0 mV); (3) Single (a single excursion that overshoots 0 mV); 4) Attempting train (several excursions but only 1 overshoots 0 mV); and (5) Train (several excursions, at least 2 of that overshoot 0 mV). Input resistance was measured from the voltage difference induced by the −1 nA current step. Spike analysis was performed on the first spike of an induced action potential train using Clampfit 11.1; threshold was determined as the peak of the 3rd differential of voltage with respect to time during the upstroke of the action potential and all other parameters are as defined extensively elsewhere [41].

Na^+^ currents in iNs were recorded using a standard voltage-step protocol, holding potential of −70 mV followed by 80 ms steps from −120 to + 80 mV in increments of 10 mV. For Na^+^ current inactivation curves, cells were stepped for 200 ms to pre-pulse voltages of between −120 and + 80 mV in 5 mV step increments before being stepped for 200 ms to the test potential of 0 mV. Cell capacitance and series resistance were measured and compensated; series resistance was compensated 60–90%. Pipette resistances were 2–5 MΩ when filled with the pipette solutions.

The normalised conductance values (G) for activation and inactivation were calculated by dividing current by the appropriate driving force, (V_c_ – E_Na_), where V_c_ = command potential, and E_Na_ =  + 66.7 mV. G/G_max_ was plotted against voltage and fitted with a Boltzmann equation using an iterative fitting routine:

$$G/G\text{max} = 1/[1 + exp(V50 - Vc)/h]$$

G_max_ is the extrapolated maximum conductance, Va_50_ and Vi_50_ are the voltages corresponding to half the maximum conductance and h is the slope factor.

### Measurement of cholesterol

Cholesterol levels were assessed using a commercial assay kit (Cat# 60723ES60; Yeasen biotechnology, Beijing, China) following the manufacturer’s instructions. In brief, cells were cultured at a density of 40,000 per well in a 6-well plate and processed to extract lipids by homogenizing them in 200 µL of isopropanol. Samples were then mixed with the reaction reagent and incubated at 37 °C for 15 min. Absorbance was measured at a wavelength of 500 nm using a spectrophotometer. Cholesterol concentrations were calculated based on a standard curve.

### Gene knockdown

siRNA targeting FDFT1 was synthesized based on the sequence provided in this paper [42], as shown in the Table 2. iNs were cultured in Neurobasal medium supplemented with B27 and neurotrophic factors at 37 °C in a 5% CO2 atmosphere. For transfection, 25 pmol of siRNA was added per well in a 6-well plate using Lipofectamine RNAiMax reagent (Cat#13,778,030; Thermo Fisher Scientific, Waltham, MA, USA) according to the manufacturer's instructions. Gene knockdown was evaluated using quantitative RT-PCR and subsequent Western blotting to assess FDFT1 expression after 1, 2 and 7 days.

**Table 2:** siRNA oligos used in this study

Gene	Sense	Anti-sense
FDFT1	5’ –GGAAGAGAUUUAUCAUAGAAU–3’	5’ –UCUAUGAUAAAUCUCUUCCAU–3’

### Statistical analysis

Electrophysiological data were analyzed using Clampfit 11.1, Microsoft Excel, and Microcal Origin 6.0 software and expressed as mean ± SEM. Other data were addressed by Graphpad prism 8.0 software and expressed as mean ± SEM. Statistical comparisons of the means within two groups were performed using Student’s t-tests, while one-way ANOVA followed by Tukey’s post-hoc test was employed to compare means across three or more groups.

## Results

### PEMF stimulation enhances the expression of synaptic proteins in Ngn2-directed iPSC-derived neurons

The effects of magnetic stimulation on cell differentiation, neurotrophic factor expression, and peripheral nerve regeneration are influenced by intensity, duration, and frequency [43]. A 2023 meta-analysis of 92 studies (1999–2019) identified magnetic flux densities of 1–10 mT as optimal for eliciting robust cellular responses [44]. In vitro experiments further demonstrate that moderate-intensity PEMF exposure (0.19–1.37 mT) significantly enhances neurite outgrowth, upregulates c-fos expression, and promotes neural-like morphological changes [45–47]. Exposure of embryonic neural stem cells to 1mT and 50 Hz electromagnetic fields (EMF) promotes neuronal maturation in vitro [43], while EMF with 75 Hz square-wave reduces the neuron/astrocyte ratio in bone marrow mesenchymal stem cells compared to lower frequencies [48]. Recent evidence suggests pulsed electromagnetic fields (PEMF) exhibit stronger bioactivity than continuous electromagnetic fields (CEMF) [44].

**Fig. 1:**
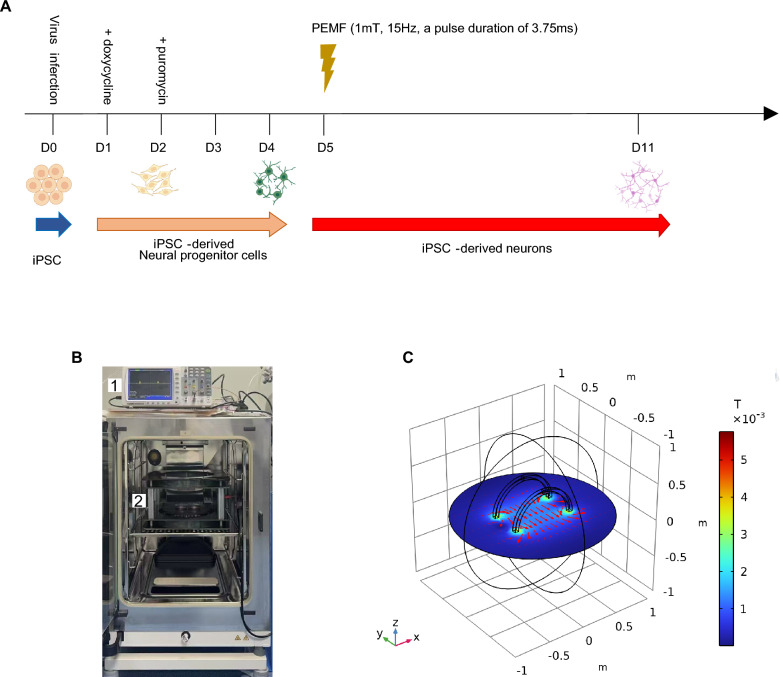
Schematic of PEMF treatment and properties of PEMF. **a** Flow diagram of iNs generation and PEMF treatment. **b** Equipment of magnetic stimulation applied on cells, which is put on the cell incubator. Digital oscilloscope revealed the pulsed electromagnetic wave with time, indicated in label 1. Cells cultured in dish or plate were put on the plateau between two coils (label 2) for PEMF stimulation. **c** COMOSL Multiphysics model of magnetic field, indicating the distribution of magnetic field between Helmholtz coils. Directions of magnetic stimulation was labeled by red arrows in plane of Z = 0. The spatial distribution of electromagnetic field intensity in this project is presented from 1 to 5 mT

Prior work has shown that human iPSCs can be converted into functional neurons that moderately express several synaptic genes within two weeks by the forced expression of Ngn2 [20]. The relative neurite length and branch points, as determined by an IncuCyte live-cell imaging paradigm, increase rapidly from iN day 5–14 but thereafter remain constant, indicating the formation of approximate neuron structural networks [49]. To select optimal stimulation approaches on regulating neuronal differentiation, we first exposed iNs to CEMF at low (15 Hz) and high (75 Hz) frequencies from iN days 5–11. Cells were plated in 96-well plates on day 4 and transferred to the magnetic stimulation system the following day. Each plate was treated for 2 h per day over 7 days (Fig. 1B). Helmholtz coils modeling was performed using the COMSOL application (Version 6.3). The results depicted the magnetic field distribution generated by the Helmholtz coils, as illustrated in Fig. 1C. It indicated the uniform direction and intensity of the magnetic field between the two coils along the Y-axis (Fig. 1C). The cultured cells were put on the platform between two coils and the intensity of magnetic fields were measured to be 1 mT when the input current was 2.9 A (Fig. 1C). Notably, 15 Hz CEMF enhanced expression of synaptophysin but not synapsin I, while 75 Hz CEMF did not show significant effect on these two synaptic markers (Supplementary Fig. 1). PEMF with identical parameters (15 Hz frequency, 1 mT intensity) significantly upregulated the expression of synapsin I and synaptophysin (Supplementary Fig. 1). Based on these findings, we selected PEMF and implemented stimulation with optimized parameters: 1 mT magnetic flux density, 15 Hz frequency, and 3.75 ms pulse duration (Fig. 1a).

**Fig. 2:**
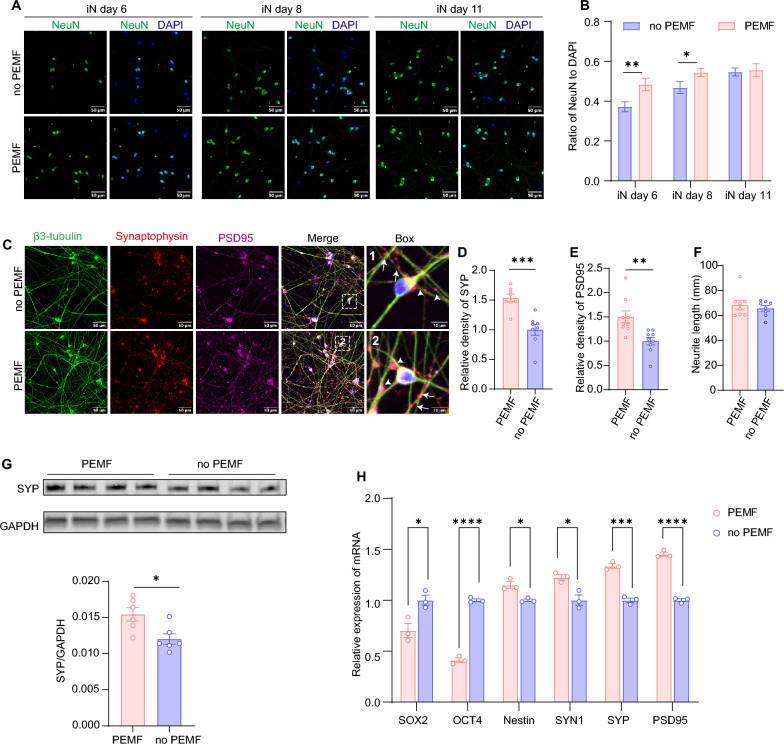
PEMF stimulation promotes the Ngn2-directed neuronal differentiation of iNs. **a** Immunocytochemical images produced by a confocal microscope showing NeuN expression in iNs with PEMF or without PEMF at different time points. ICC was performed by anti-NeuN (*green*) and DAPI (*blue*). Scale bar: 50 μm. N = 4 for both groups **b** Ratio of NeuN to DAPI from panel A was calculated and presented in a histogram plot. **c** Representative images of iNs with or without PEMF stimulation for 7 days. ICC was performed with DAPI (*blue*), anti-β3 tubulin (*green*), anti-synaptophysin (*red*) and anti-PSD95 (*purple*). Higher magnification of the boxed region of panel A shows the expression of synaptophysin (*arrow*) and PSD95 (arrowhead) associated with the neurite. Scale bars: 50 (left) and 10 μm (right) **d** and **e**. Quantitative analysis of relative density shows that PEMF significantly increases the expression of synapophysin and PSD95, n = 4 in both groups. **f** Histogram plots of neurite length stained by β3-tubulin showing no significant difference between PEMF and no PEMF group, n = 4 for both groups. **g** Western blot analysis of synaptophysin (SYP) in iNs with or without 7-day PEMF stimulation. GAPDH was used as internal control. The uncropped blot is shown in supplementary Fig. 6A. **h** Real time qPCR analysis of stem cell markers (*SOX2 and OCT4*), neural development marker (*Nestin*) and synaptic markers (*SYN1, SYP and PSD95*) in iNs with or without PEMF stimulation for 7 days, n = 3 for both groups. Results are presented as the mean ± SEM from three independent experiments. Numbers of sample mean the wells of cultured cells. Treatments were examined by student’s t-test and significant differences are denoted as *P < 0.05, **P < 0. 01 and ***P < 0.001. ns indicates not significant

Beginning on day 5 of iN differentiation, cells were exposed to daily 2-h PEMF treatments or maintained as untreated controls, then fixed for analysis on iN day 6, 8, and 11. Neuronal differentiation was assessed using NeuN immunostaining (to identify mature neurons) and DAPI (to quantify total cell nuclei). PEMF-treated iNs exhibited a significantly higher NeuN^+^/DAPI^+^ ratio compared to controls at iN day 6 and 8, though this difference disappeared by iN day 11 (Fig. 2a, b). Quantification of neurite length (labeled with β3-tubulin) and live-cell imaging using the Incucyte system both demonstrated that PEMF treatment had no significant effect on neurite outgrowth and branch points of neurite in iNs during the entire stimulation period (Fig. 2f and Supplementary Fig. 2). However, PEMF-treated cells showed markedly elevated expression of synaptic proteins synaptophysin and PSD95 relative to neurite density at iN day 11 (Fig. 2c–e). Similar results were observed by immunoblotting which confirmed that PEMF promoted the expression of synaptophysin (Fig. 2g and Supplementary Fig.  6 A). qPCR measurements of the expression of the stem cell markers *SOX2* and *PAX6* revealed a decrease in pluripotency of iNs, suggesting that iNs can reach terminal differentiation more efficiently upon PEMF stimulation (Fig. 2h). In contrast, expression of the genes related to neural development (*Nestin*) and synapse formation (*SYN1*, *SYP*, and *PSD95*) were all upregulated by PEMF stimulation (Fig. 2h). These findings suggest that PEMF accelerates early-stage neuronal differentiation and enhances synaptic protein expression following sustained treatment.

### PEMF stimulation boosts the spontaneous calcium oscillations and action potentials of iNs

**Fig. 3:**
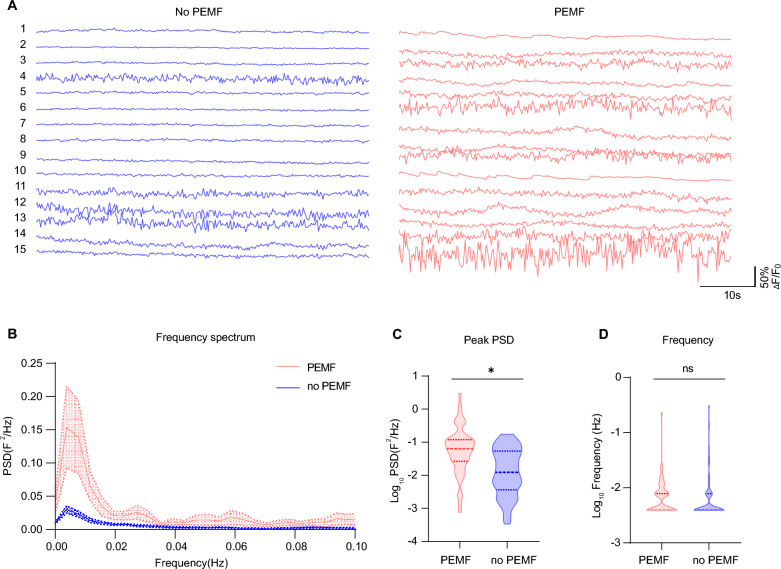
PEMF stimulation potentiates the spontaneous calcium oscillations of iNs. **a** Representative Ca^2+^ traces of neurons with (right panel) or without (left panel) PEMF stimulation. Recordings were performed for 60 s and data at each time point was normalized to baseline signal (F_0_). *Scale bar* indicated 10 s on x-axis and 50% of normalized fluorescence intensity on y-axis. **b** Power spectrum analysis of spontaneous calcium oscillations of iNs with (*red*, n = 52) or without (*blue*, n = 57) PEMF. **c** Peak of power spectra density (PSD) was significantly higher in PEMF group than the no PEMF group. Results are presented as the mean ± SEM from three independent experiments. Treatments were examined by student’s t-test and significant differences are denoted as *P < 0.05. **d** Frequency of calcium wave showed no significant difference between the two groups. ns indicates not significant

Spontaneous calcium oscillations, also known as calcium transients, are observed in various cell types including differentiated neurons and NPCs. In the initial stages, NPCs exhibit frequent and high-amplitude spontaneous calcium oscillations that are crucial for the cellular signaling and the establishment of early neural identity. After exposure to PEMF stimulation for 7 days, iNs exerted stronger spontaneous calcium oscillations than non-treated cells (Fig. 3a). Power spectral density (PSD) is a physical quantity that characterizes the power distribution of a signal over frequency, in our case is a measurement of spontaneous neuronal calcium waves in the neurons. The frequency spectrum revealed a significant enhancement of PSD across low-frequency ranges (0–0.1 Hz) in the PEMF group (*n* = 52) compared to the no PEMF group (*n* = 57) (Fig. 3b, c). These data indicated that one-week of PEMF stimulation at an early stage of neuronal differentiation can enhance the spontaneous calcium activity of iNs.

**Fig. 4:**
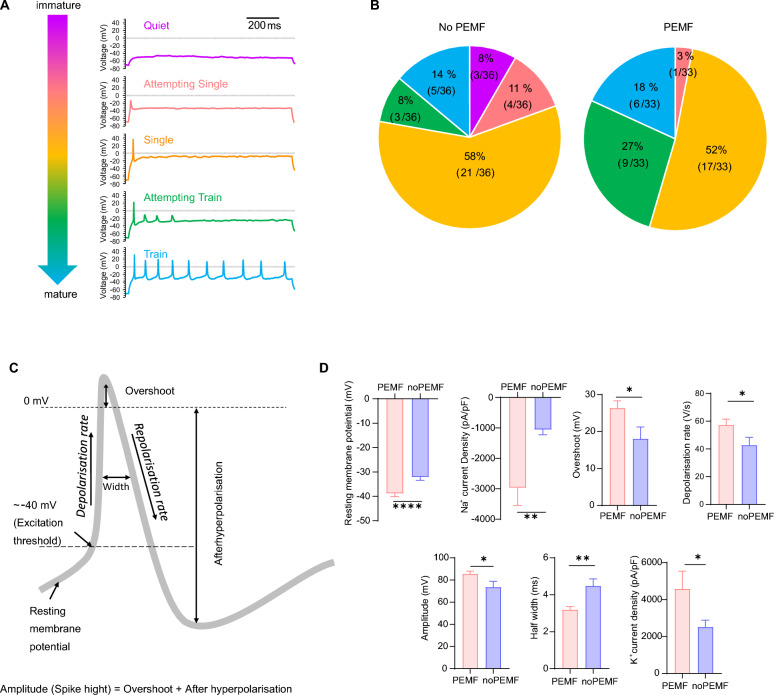
Effects of PEMF on the action potentials and membrane property of iNs. **a** Traces of current-clamp recordings for iNs exemplifying different model activities: no induced action potential (Quiet—*magenta*); incomplete initiation of single action potential (Attempting single—*red*); single action potential (Single—*orange*); single action potential with incomplete initiation of action potential trains (Attempting train—*green*); and train of induced action potentials (Train—*blue*). **b** Pie charts displaying percentage and proportion of various types of neuronal activity (Quiet, Attempting single, Attempting train and Train) in iNs with or without 7-day PEMF treatment (n = 36 in no PEMF group, n = 33 in PEMF group). **c** Anatomy of neuronal action potential. (**D**) Amplitude and kinetic parameters of induced action potentials (iAP) in iNs showing significant difference between the two groups: no PEMF (*blue*); PEMF (*red*). All data are shown as mean ± SEM from two batches of independent experiment. Treatments were examined by student’s t-test and significant differences are denoted as *P < 0.05, **P < 0.01 and ****P < 0.0001

In the next series of experiments, we assessed the non-stimulated and stimulated electrical activity of PEMF *vs*. no PEMF iNs by using patch-clamp electrophysiology as it was performed in the previous studies (Fig. 4a) [50, 51]. Recording of V_m_ in the iNs displayed “Quiet” and “Spontaneous” behaviour in both no PEMF and PEMF groups: in no PEMF group 97% (36 out of 37 neurons) were “Quiet” and in PEMF 3% (1 out of 36 neurons) were “Spontaneous”, whereas in PEMF group 94% (32 out of 34 neurons) were “Quiet” and in PEMF 6% (2 out of 34 neurons) were “Spontaneous” (Supplementary Table 1). In the induced activity characteristics, no PEMF iNs displayed all five types of functioning: 8% (3 out of 36 neurons) were “Quiet”, 11% (4 out of 36 neurons) were “Attempting Single”, 58% (21 out of 36 neurons) were “Single”, 8% (3 out of 36 neurons) were “Attempting Train” and 14% (5 out of 36 neurons) were “Train”. PEMF-administrated iNs had only four types of performance with no “Quiet” neurons, 3% (1 out of 33 neurons) were “Attempting Single”, 52% (17 out of 33 neurons) were “Single”, 27% (9 out of 33 neurons) were “Attempting Train” and 18% (6 out of 33 neurons) were “Train” (Fig. 4b and Supplementary Table 2). Taken together the protocols of noninduced and induced neuronal activity shows more mature functional properties of PEMF-administrated iNs *vs*. no PEMF iNs had bigger number of spontaneously active iNs (6% in PEMF vs. 3% in no PEMF) and bigger number of iNs with genuine action potentials (“Single + Attempting Train + Train”: 97% in PEMF *vs*. 80% in no PEMF).

We compared the passive cellular properties and spike characteristics of no PEMF and PEMF-treated iNs recorded in the current-clamp mode. We observed the significant differences in several parameters: the average resting V_m_ for the no PEMF group was −32.1 ± 1.3 mV (*n* = 37), compared to −38.8 ± 1.3 mV (*n* = 34) in the PEMF group (*P* < 0.001). The average overshoot of the iAP for the no PEMF group was 18.0 ± 3.2 mV (*n* = 29), whereas it was 26.3 ± 2.0 mV (*n* = 32) in the PEMF group (*P* < 0.05). The average amplitude of the iAP was 73.6 ± 5.1 mV (*n* = 29) in the no PEMF group compared to 85.5 ± 2.5 mV (*n* = 32) in the PEMF group (*P* < 0.05). The average depolarization rate of the iAP was 42.7 ± 5.7 V/s (*n* = 29) in the no PEMF group, while it was 57.3 ± 4.2 V/s (*n* = 32) in the PEMF group (*P* < 0.05). Lastly, the average half-width of the iAP in the no PEMF group was 4.5 ± 0.4 ms (*n* = 29), compared to 3.2 ± 0.2 ms (*n* = 32) in the PEMF group (*P* < 0.01) (Fig. 4d and Supplementary Table 3).

**Fig. 5:**
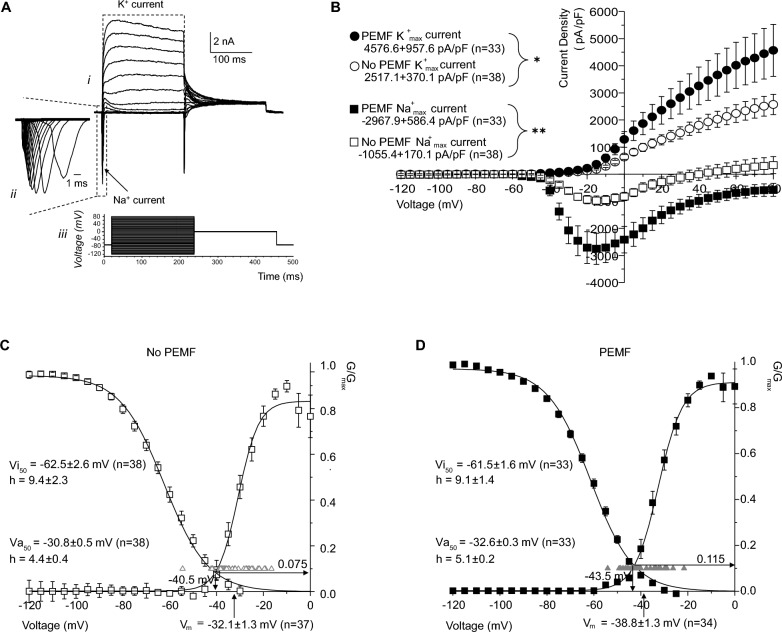
Electrophysiological characterization of Na⁺ and K⁺ currents in iNs with or without PEMF stimulation. **a** Whole-cell macroscopic exemplar traces of macroscopic inward sodium (Na^+^) and outward potassium (K^+^) voltage-gated transmembrane currents recorded from iNs (i); expanded inset showing inwards spikes corresponding to activating Na^+^ currents (ii); voltage-step activation/inactivation protocol with holding potential −70 mV, voltage range from −120 mV to 80 mV with 5 mV step increment (iii). **b** Current–voltage (I-V) relationships for peak Na⁺ and K⁺ currents in neurons exposed to PEMF (filled symbols) and no PEMF (open symbols). The PEMF group (n = 33) shows significantly larger K⁺ currents compared to no PEMF group (n = 38), while Na⁺ currents are significantly reduced in the PEMF. Values are shown as mean ± SEM. **c** and **d** Mean activation and inactivation characteristics of normalized conductance (G/G_max_) of whole-cell Na^+^ currents recorded in 7-day PEMF and no PEMF treated iNs. Mean activation and inactivation curves of Na^+^ current G/G_max_ recorded in iNs with (*filled squares*) and without (*open squares*) PEMF treatment. Individual resting membrane potential values are shown by the upward triangles, availability window maxima is shown by downward arrow and mean resting V_m_ value is shown on the x-axis by upward arrow. Va_50_: Voltages of half maximal action. Vi_50_: Half maximal inactivation. The h factors: mean crossing points (downward arrows) number of cells recorded for each group (n). All data are shown as mean ± SEM from two batches of independent experiment. Treatments were examined by student’s t-test and significant differences are denoted as *P < 0.05 and **P < 0.01

To understand the basis of the enhanced excitability observed in the PEMF-treated iNs, we recorded transmembrane voltage-gated inward sodium (Na^+^) and outward potassium (K^+^) current densities using voltage-clamp mode. The average maximal current density amplitude for Na^+^ voltage-gated channels in the no PEMF group was −1055.4 ± 170.1 pA/pF (*n* = 38), compared to −2967.9 ± 586.4 pA/pF (*n* = 33) in the PEMF group (*P* < 0.01). For K^+^ voltage-gated channels, the average maximal current density amplitude was 2517.1 ± 370.1 pA/pF (*n* = 38) in the no PEMF group, versus 4576.6 ± 957.6 pA/pF (*n* = 33) in the PEMF group (*P* < 0.05) (Fig. 5b and Supplementary Table 3), indicating a faster maturation rate for the PEMF-treated iNs.

The activation and inactivation profiles of the voltage-activated Na^+^ currents in both no PEMF and PEMF groups showed no changes in the availability current window. However, the G/G_max_ maxima in the PEMF group was larger than in the no PEMF group (0.115 vs. 0.075). There was no significant difference between the mean half-activation and half-inhibition voltages (*V*_a50_—*V*_i50_), which were 31.7 ± 2.1 mV (*n* = 38) for the no PEMF group and 28.9 ± 1.3 mV (*n* = 33) for the PEMF group, respectively (Fig. 5c, d). These data suggest that the improved excitability parameters in the PEMF-treated iNs are attributed to the up regulation of transmembrane Na^+^ voltage-gated conductance, which is responsible for generating neuronal action potentials.

### PEMF stimulation promotes neuronal maturation by up-regulating cholesterol biosynthesis pathway

**Fig. 6:**
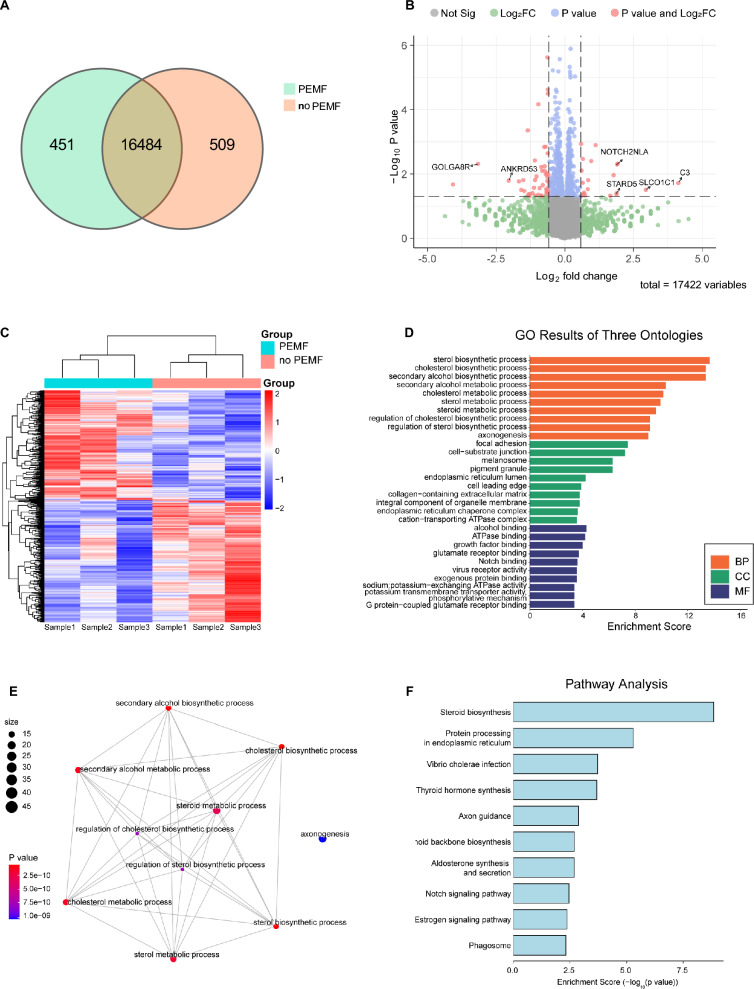
Transcriptomic analysis of differentially expressed genes (DEGs) and associated biological processes and pathways in iNs with or without PEMF stimulation. **a** Venn diagram showing genes solely expressed in no PEMF group (*yellow circle*) or PEMF group (*green circle*), and genes commonly expressed in both groups (intersection). **b** Volcano plot of DEGs identified in PEMF vs. no PEMF groups. DEGs with log_2_ fold change > 1.2 and *P* < 0.05 are shown in red. **c** Heat map of hierarchical clustering analysis showing the expression patterns of DEGs between PEMF and no PEMF groups. Up regulated and down regulated genes are shown in red and blue respectively. **d** Gene Ontology (GO) enrichment analysis of DEGs following three criteria: Biological Process (BP), Cellular Component (CC) and Molecular Function (MF). The x-axis represents the enrichment score and the y-axis lists the significantly enriched GO terms (*P* < 0.05). The most enriched processes include sterol biosynthetic process, axonogenesis and cholesterol metabolism. **e** Network plot of the top enriched GO terms related to BP. Nodes represent individual GO terms with size corresponding to the number of genes involved. The color gradient represents *P* value and significance. Key processes such as sterol biosynthetic process and cholesterol metabolic process are prominently interconnected. **f** KEGG pathway analysis of DEGs. The x-axis shows the enrichment score (−log_₁₀_ *P* value) and the y-axis shows top pathways, among which steroid biosynthesis, protein processing in the endoplasmic reticulum and axon guidance are the most significantly enriched. All groups have three biological replicates

To investigate the mechanism by which PEMF enhanced neuronal maturation, we conducted RNA sequencing on iNs with or without PEMF stimulation. Venn diagram analysis revealed 16,484 common genes and 960 differentially expressed genes (DEGs) in the two groups, among which 451 genes only appeared in the PEMF group and 509 genes appeared in the no PEMF group (Fig. 6a). Subsequent analysis of the top genes using volcano plots yielded candidates that might be associated with PEMF-induced neuronal differentiation (Fig. 6b). PEMF stimulation up-regulated 338 genes and down-regulated 373 genes in iNs (*P* < 0.05, fold-change > 1.2) (Fig. 6b). The top upregulated genes suggested that PEMF has a strong association with neuronal development (*C3* and *NOTCH2NLA*) and sterol metabolism, including bile acid and cholesterol transport (*STARD5* and *SLCO1C1*) (Fig. 6b). The downregulated genes revealed that PEMF may inhibit spindle assembly (*GOLGA8R* and *ANKRD53*) and therefore manipulate cell proliferation (Fig. 6b). Hierarchical clustering analysis revealed that three batches of iNs from PEMF and no PEMF group clustered together within each respective group (Fig. 6c).

Gene Ontology (GO) analysis of the identified transcripts suggested that PEMF stimulation enhanced several biological processes, including the biosynthesis and metabolism of sterols, cholesterol and secondary alcohols, as well as axonogenesis (Fig. 6d, e). GO cellular component analysis revealed significant enrichment of DEGs in the endoplasmic reticulum (ER) lumen and the ER chaperone complex. These data suggest a strong correlation of PFMF stimulation and ER-related biosynthesis, since the biosynthesis of sterols and cholesterol extensively occurs in the ER of nucleated cells. In addition, GO molecular function analysis suggested that PEMF significantly enhanced the activities of ATPase binding, growth factor binding and glutamate receptor binding, all of which are highly associated with neuronal differentiation (Fig. 6d). Similar findings were observed from KEGG pathway analysis, which indicated that pathways related to steroid biosynthesis and protein processing in the ER are significantly enriched upon PEMF stimulation (Fig. 6f). Collectively, the transcriptomic analysis suggest that sterol biosynthesis is likely to play a central role in the enhancement of neuronal development by PEMF stimulation.

**Fig. 7:**
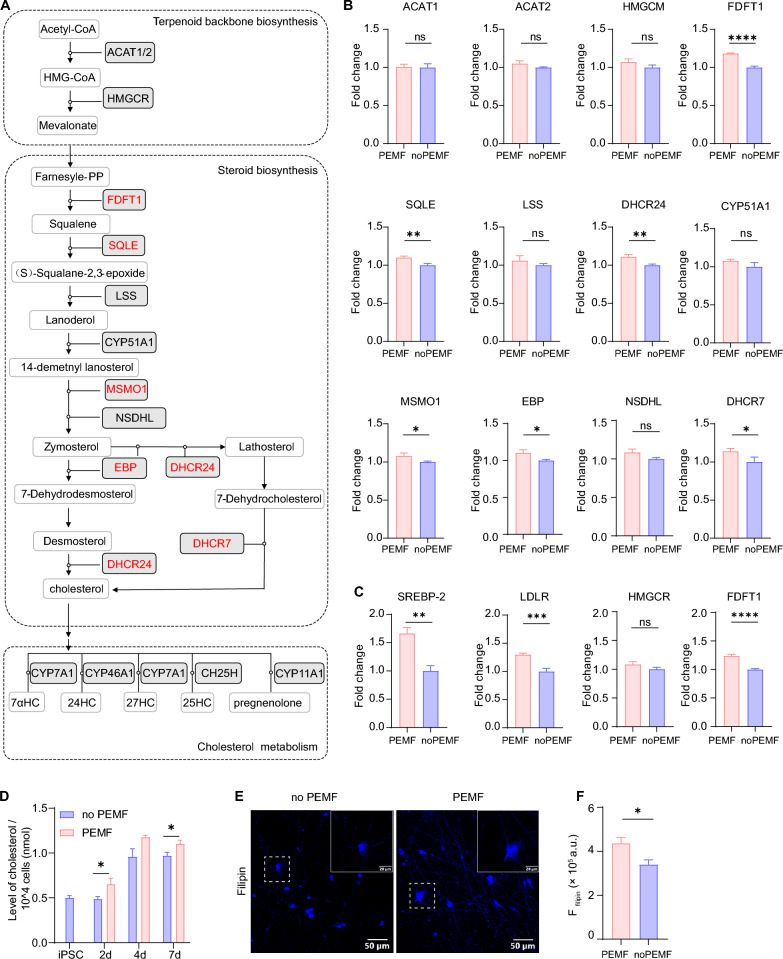
PEMF stimulation up-regulates the biosynthesis of cholesterol during the differentiation process of iNs. **a** Schematic illustration of cholesterol biosynthesis and metabolism, including terpenoid backbone biosynthesis, steroid biosynthesis and cholesterol metabolism. **b** Relative gene expression levels in the cholesterol biosynthesis pathway quantitated by RNA-seq, n = 3 for both groups. **c** Real-time qPCR analysis indicated higher expression of genes related to cholesterol homeostasis (*SREBP, FDFT1*) and metabolism (*LDLR*) in the PEMF group, no significant difference in expression of *HMGCR* between groups. n = 3 for both groups. **d** The level of cholesterol was measured at different time points of iNs (2d: iN day 4, 4d: iN day 6, 7d: iN day 11), with findings showing that PEMF enhanced the biosynthesis of cholesterol in iNs. n = 3 for both groups. **e** Representative immunostaining images of iNs show elevated filipin in the PEMF-treated group. Scale bars: 50 and 20 μm (box). **f** Quantitative analysis of filipin intensity in confocal images, suggesting PEMF increases the biosynthesis of cholesterol, n = 4 for both groups. All data are shown as mean ± SEM. All data, except for the RNA-seq data, were from two or three independent experiments. The sample numbers refer to the wells of cultured cells used in these experiments. Treatments were examined by student’s T-test and significant differences are denoted as *P < 0.05, **P < 0.01, ***P < 0.001 and ****P < 0.0001. ns denotes not significant

Sterol biosynthesis is a metabolic process involved in terpenoid backbone biosynthesis and steroid biosynthesis, which converts acetyl-CoA into cholesterol and other sterols. Cholesterol is a critical precursor for steroid hormones (e.g., pregnenolone) and bile acids (Fig. 7a). Bile acids can promote neurite outgrowth of NSC-34 neurons and have protective effects in neurogenesis [52]. To identify key genes underlying PEMF-enhanced neuronal maturation, we analyzed expression levels of enzymes critical to the cholesterol biosynthesis pathway (Fig. 7a). PEMF stimulation significantly upregulated genes clustered in steroid biosynthesis, including FDFT1, SQLE, DHCR24, MSMO1, EBP, and DHCR7 (Fig. 7b). In contrast, expression of genes encoding upstream terpenoid-backbone biosynthesis enzymes—acetyl-CoA acetyltransferase (ACAT1, ACAT2) and HMG-CoA reductase (HMGCR)—showed no significant differences between PEMF-treated and control groups (Fig. 7b). Sterol regulatory element-binding protein 2 (SREBP-2) is a master regulator of cholesterol biosynthesis and upregulates key enzymes such as FDFT1 and HMGCR [53]. The Low-Density Lipoprotein Receptor (LDLR) mediates cellular cholesterol uptake [54]. Our qPCR analysis revealed elevated mRNA levels of SREBP-2, LDLR and FDFT1 in PEMF-treated cells, but no significant difference of HMGCR was observed (Fig. 7c). These data suggest that PEMF stimulation enhances cholesterol biosynthesis and metabolic turnover independently of the mevalonate pathway.

To determine whether PEMF has an early metabolic effect, we then assessed cholesterol levels at multiple time points in the neuronal differentiation process. At 2 days with PEMF stimulation, cholesterol levels in iNs remained comparable to undifferentiated iPSCs but were significantly elevated in PEMF-treated groups. Cholesterol production in iNs increased progressively over time, plateauing at iN day 8. PEMF stimulation exhibited a sustained trend toward enhancing cholesterol biosynthesis throughout the differentiation period, though the difference between PEMF-treated and unstimulated groups at the 4-day time point (iN day 8) did not reach statistical significance (Fig. 7e). These findings suggest PEMF has an early-stage metabolic influence on cholesterol production during neuronal differentiation, consistent with prior observations of temporally regulated cholesterol accumulation during neural stem cell differentiation into neurons [55].

### Pharmacological inhibition or genetic knockdown of FDFT1 abolishes the PEMF-induced enhancement of neuronal maturation

Squalene synthase (farnesyl diphosphate farnesyltransferase, FDFT1) is a key enzyme that catalyzes squalene production and sterol synthesis at the gateway of the cholesterol biosynthesis pathway (Fig. 7a). Numerous studies have shown that cholesterol is indispensable for neuronal development. Embryos lacking functional squalene synthase exhibit severe growth retardation and neural tube closure defects [56]. Neurons from *FDFT1* mutant mice display stunted neurite outgrowth and reduced dendritic complexity [57]. Similarly, pharmacological inhibition of squalene synthase in immature Purkinje cells suppresses dendritic branching during development [58]. Collectively, these findings suggest *FDFT1* is essential in cholesterol synthesis during neuronal maturation, particularly in neurite extension and dendritic arborization. Our data suggests that *FDFT1* is significantly upregulated upon PEMF stimulation, positioning it as an important candidate among DEGs mediating PEMF’s pro-maturational effects (Fig. 7b).

**Fig. 8:**
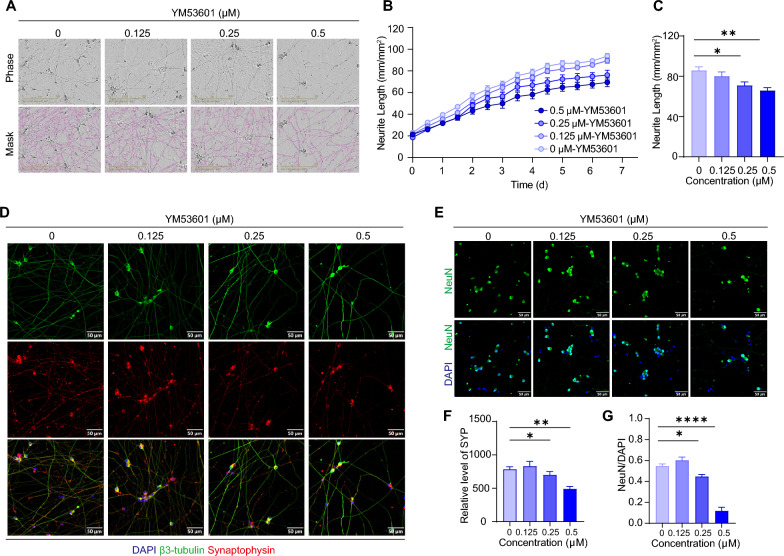
Pharmaceutical blockage of FDFT1 inhibits neuronal development of iNs. **a** Representative images and identified neurite of iNs treated with YM53601, an inhibitor of FDFT1. The top panel shows phase-contrast images and the bottom panel shows composite images of the identified neurons (*purple*) and phase-contrast field. Scale bar: 200 μm. **b** Time-course plots illustrating the neurite outgrowth of iNs treated with different concentrations of YM53601. **c** Histogram plots of normalized neurite length derived from the last point in panel C, n = 6 for both groups. **d** Immunocytochemical characterization of synaptophysin (*red*), β3-tubulin (*green*) and DAPI (blue) in iNs treated with YM53601 at various concentrations. Scale bar: 50 μm, n = 3 for both groups. **e** Immunocytochemical characterization of NeuN (green) and DAPI (blue) in iNs treated with YM53601 at various concentrations. *Scale bar*: 50 μm, n = 4 for both groups. **f** Quantitation of synaptophysin expression in iNs treated with YM53601 for 7 days. **g** Ratio of NeuN to DAPI in iNs treated with YM53601 for 7 days. All data are shown as mean ± SEM and two batches of individual experiment have been performed. Numbers of sample mean the wells of cultured cells. Treatments were examined by one-way ANOVA and significant differences are denoted as *P < 0.05, **P < 0.01 and ****P < 0.0001

To determine the functional role of *FDFT1*, we first tested whether its squalene synthase activity is essential for neuronal differentiation under the physiological condition. YM53601 is a small molecule that inhibits squalene synthase and potently suppresses cholesterol biosynthesis across several species. Treatment with YM53601 suppressed neurite growth of iNs in both concentration- and time-dependent manners, indicating that FDFT1 is critical in the formation of neuronal structural network (Fig. 8a–c). Immunostaining futher showed that prolonged YM53601 treatment (7 days) progressively diminished NeuN expression in iNs in a concentration-dependent manner (Fig. 8e–g). The expression of synaptophysin, a synaptic vesicle marker, was also dose-dependently reduced by YM53061 (Fig. 8d and f). These data suggest that cholesterol biogenesis is required for both neuronal differentiation and synapse formation.

**Fig. 9:**
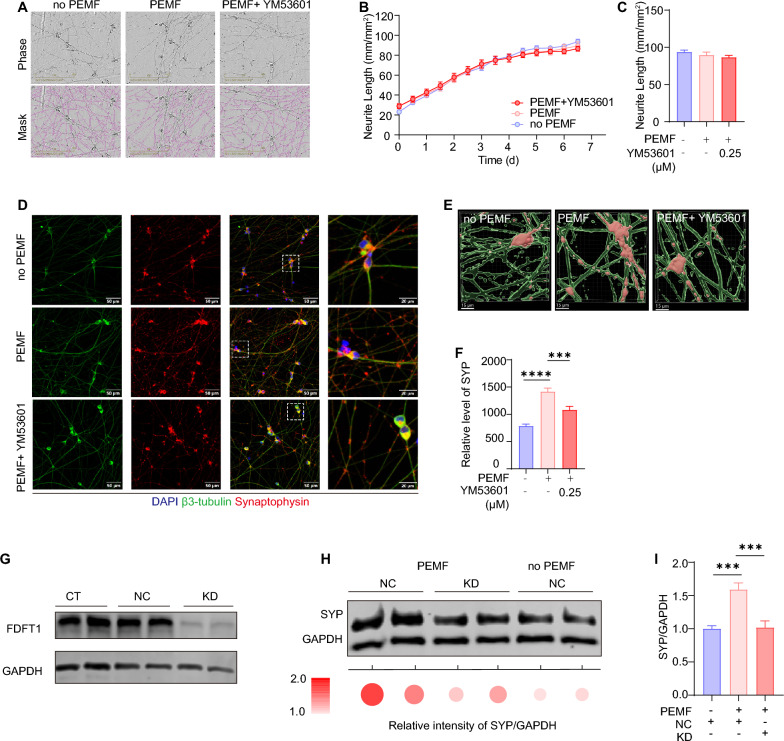
Pharmaceutical blockage of FDFT1 and *FDFT1*-knocking down both abolish the effect of PEMF-promoted the neuronal maturation. **a** Representative images of iNs captured by the IncuCyte live-cell system in three groups: no PEMF, PEMF, and PEMF combined with YM53601 treatment. Scale bar: 200 μm. **b** Time-course plots showing neurite outgrowth of iNs from the three groups in panel A. **c** Histogram plots of normalized neurite length derived from the last time point in panel B, n = 6 for all groups. **d** Immunocytochemical images produced by a confocal microscope showing synaptophysin expression in iNs across the three groups. Scale bar: 50 μm. **e** A 3D reconstruction of iNs, based on Z-stack confocal images, displaying the location and expression of synaptophysin in iNs of the three groups. Scale bar: 10 μm. **f** Quantitative analysis of synaptophysin in iNs from the three groups on the 7th day of neuronal differentiation, n = 4 in all groups. **g** Western blot analysis detected the protein level of FDFT1 in three groups on iN day 11: control (CT), *FDFT1*-knockdown (KD) and negative control (NC) at different time points. **h** Western blot analyzing synaptophysin (SYP) of iNs in response to *FDFT1*-knockdown under the PEMF stimulation or not. GAPDH was used as internal control, n = 3 in all groups. **i** Histogram plots of SYP expression from panel H. All data are shown as mean ± SEM and two or three batches of individual experiment have been performed. Numbers of sample mean the wells of cultured cells. Treatments were examined by one-way ANOVA and significant differences are denoted as *** *P* < 0.001 and ****P < 0.0001

Having confirmed the critical role of *FDFT1* in physiological neuronal differentiation, we went on to examine whether *FDFT1* activity is required in PEMF-promoted neuronal maturation. Continuous PEMF stimulation without YM53061 treatment over a 7-day period had no effect on neurite outgrowth of iNs (Fig. 9a–c) but significantly enhanced the expression of synaptophysin (Fig. 9d–f). This enhancement is consistent with increased PSD95 immunostaining signals (Fig. 2), further supporting PEMF’s effects on synaptic maturation. While no significant difference in neurite outgrowth was detected under PEMF stimulation, PEMF exposure restored normal neurite outgrowth in iNs with a continuous YM53061 treatment for 7 days (Fig. 9a–c and Supplementary Fig.  3 A, 3 C and 3D). This is consistent with a prior study that cholesterol supplementation had minimal effects on control neurons, but fully rescued axon length and dendritic complexity in neurons with *FDFT1* mutants [57]. The ability of PEMF to increase synaptophysin expression levels was significantly attenuated when YM53601 was present during the one-week stimulation period (Fig. 9d–f and Supplementary Fig. 3B and 3E). Similarly, PEMF’s effect on elevating NeuN levels in iNs during early neuronal differentiation was abolished following YM53601 application (Supplementary Fig. 4).

To further validate the role of FDFT1 in PEMF-promoted neuronal differentiation and maturation, we specifically downregulated FDFT1 expression in iNs using RNA interference. Real-time PCR indicated that siRNA rapidly and efficiently reduced *FDFT1* mRNA levels within 24 h (Supplementary Fig.  3 F), and western blot analysis confirmed sustained suppression of FDFT1 protein in iNs by the 7th day of knockdown (Fig. 9g and Supplementary Fig. 6B). PEMF stimulation failed to increase synaptophysin levels in FDFT1-knockdown iNs, whereas control iNs exhibited markedly elevated synaptophysin expression following PEMF treatment (Fig. 9l and Supplementary Fig. 6C-E). These data suggest that continuous PEMF stimulation enhances synaptophysin expression independently of neurite outgrowth, and that the squalene synthase activity of *FDFT1* plays an essential role in this synaptic modulation process.

To determine whether the effects of PEMF are sustained or transient, we measured synaptophysin levels in iNs at extended time points following a 7-day PEMF stimulation protocol. Beginning on day 5 of iN differentiation, cells were either exposed to PEMF or maintained as untreated controls for 7 days (iN day 5–11) and subsequently cultured without stimulation until iN day 18 and 25. At iN day 18, synaptophysin expression in PEMF-treated iNs showed a modest elevation compared to untreated controls, though this difference did not reach statistical significance (Supplementary Fig. 5A-B). By iN day 25, synaptophysin levels became indistinguishable between the two groups (Supplementary Fig. 5C-D). These results indicate that PEMF enhances neuronal differentiation and synaptic maturation primarily during early neuronal maturation phases, with its effects not sustaining over prolonged culture periods.

## Discussion

Neural stem cells (NSCs) are valuable tools for studying neurodevelopment and sources for cell-based therapies targeting neurological disorders [59–61]. A critical challenge lies in determining the extent to which transplanted NSCs differentiate into functionally integrated neurons and establish synaptic connections within host neuronal circuits. Recent advances demonstrate that biophysical stimulation—including mechanical, electrical, or electromagnetic cues—can profoundly enhance the differentiation and functional maturation of stem cell populations [62, 63]. In this study, we report that PEMF accelerated early-stage differentiation of human cortical neurons without altering neurite outgrowth and enhanced synaptic maturation after sustained stimulation. PEMF-treated neurons displayed heightened spontaneous calcium signaling and improved functional maturation, including enhanced excitability, action potential kinetics, and voltage-gated ion channel activity. These effects are mechanistically linked to the activation of FDFT1-mediated cholesterol biosynthesis. with FDFT1 (squalene synthase) as a central regulator. Pharmacological inhibition or genetic knockdown of FDFT1 abolished PEMF-induced neuronal differentiation and synaptic maturation.

Prior studies have demonstrated that magnetic field exposure enhances neuronal differentiation by promoting synaptic integrity and neurite outgrowth [64, 65]. For example, extremely low-frequency EMF were shown to accelerate neurite elongation and synaptic maturation in embryonic NSCs [66]. In contrast, PEMF stimulation does not significantly alter neurite length in differentiating iNs. Intermittent theta burst stimulation (iTBS), a subtype of PEMF, selectively increases PSD-95 and synaptophysin expression in iNs without affecting neurite morphology [67]. Our data revealed that PEMF-treated iNs exhibited a significantly higher NeuN^+^/DAPI^+^ ratio compared to controls at early stage, but this difference disappeared by iN day 11 (Fig. 2a, b). PEMF treatment had no significant effect on neurite outgrowth in iNs during the entire stimulation period (Fig. 2f and Supplementary Fig. 2). These discrepancies highlight the critical influence of EMF parameters (e.g., frequency, intensity, duration) on neuronal maturation, with distinct subtypes eliciting divergent effects on structural outcomes.

Prior studies demonstrate that morphological maturation of iPSC-derived neurons precedes functional maturation. For example, in iPSC-derived glutamatergic and GABAergic neurons, somatic growth plateaus between days 7–12 of differentiation, followed by a reduction in soma area after day 12. Similarly, neurite length and branching peak by day 9 before declining, whereas functional synapse formation emerges only after day 23 [68]. This sequential timeline, where neurite arborization precedes synaptic and functional maturation, is in accord with the biological principle that physiological neuronal activity requires an established structural foundation. Our findings, consistent with earlier work, reveal that PEMF stimulation selectively enhances NeuN expression during early neuronal differentiation and potentiates synaptic protein expression and electrophysiological properties following sustained treatment.

The accelerated synaptic development induced by PEMF correlated with enhanced electrophysiological maturation in differentiated neurons. Our data demonstrate that a 7-day PEMF regimen significantly increased spontaneous calcium oscillations in iNs, particularly amplifying the amplitude of these oscillations. Calcium fluctuations are known to activate downstream signaling cascades that drive transcriptional programs critical for neuronal differentiation [69]. The ability of PEMF to elevate calcium activity is consistent to prior observations in MSCs, where it promoted osteogenesis and mitigated radiation-induced bone loss via similar calcium-dependent mechanisms [32]. In NSCs, electromagnetic fields enhance calcium influx by upregulating L-type calcium channels (Cav1), thereby accelerating neuronal differentiation [66]. Functional neuronal maturation was further evidenced by PEMF-induced improvements in electrophysiological properties. Electrical stimulation paradigms have been shown to elevate the proportion of neurons exhibiting spontaneous activity and augment resting membrane potential [62]. Similarly, static magnetic stimulation enhances input resistance and promotes repetitive action potential firing in mouse neural progenitor-derived neurons [70]. Consistent with these findings, our patch-clamp recordings revealed that PEMF-treated iNs exhibited a higher proportion of cells capable of generating action potentials, along with improved spike kinetics (e.g., faster rise time) and increased amplitudes of both inward Na⁺ and outward K⁺ currents compared to untreated controls (Fig. 3). These results suggest that PEMF-driven functional maturation coincides with synaptic protein upregulation, likely mediated by calcium-dependent pathways.

To elucidate the molecular mechanism underlying PEMF-promoted neuronal differentiation, we performed transcriptomic analysis of iNs with and without PEMF stimulation. GO analysis and KEGG pathway analysis of DEGs revealed significant enrichment in cholesterol biosynthesis pathways. Prior studies have shown that cholesterol plays a vital role in neuronal development and differentiation, providing structural and functional support for membrane formation, signaling, and synaptic maturation [71, 72]. NPCs and differentiating neurons require sufficient cholesterol biosynthesis for their membrane formation and signaling transduction during development [55]. Glial cells, especially astrocytes, produce cholesterol and supply it to neurons through apolipoproteins to support synaptic function [73, 74]. Human cytomegalovirus suppresses SREBP2 and thereby reduces intracellular cholesterol levels and blocks neuronal differentiation [75]. Similarly, mutations in DHCR7, a gene critical for the final step of cholesterol synthesis, cause Smith-Lemli-Opitz syndrome (SLOS), a neurodevelopmental disorder characterized by brain malformations and impaired neural differentiation [76]. Piezo1-knockout ESCs exhibit reduced neural differentiation due to cholesterol deficiency, while FDFT1-knockout mice display smaller brain volumes, thinner neuroepithelia, and fewer neurons[77, 78]. Moreover, cholesterol is a precursor for steroid hormones and neurosteroids, which regulate neural differentiation by modulating neurotransmitters and excitabilities [79]. Collectively, evidence from genetically modified models and clinical studies indicates that impaired cholesterol biosynthesis is a driver of dysfunctional neurogenesis. In the present study, we found that treatment with YM53601 suppressed neurite growth, reduced the expression of synaptophysin and NeuN (Fig. 8). These findings verify the importance of the cholesterol biosynthesis pathway in neuronal differentiation and synapse formation.

Numerous studies have investigated the interplay between magnetic fields and cholesterol metabolism [80–83]. Deficiencies in cholesterol-related proteins or lipid transporters (e.g., APOE, LDLR) disrupt cholesterol biosynthesis during neuronal differentiation, highlighting its functional importance in the process [73]. Our work demonstrated that FDFT1—a key upstream enzyme that initiates steroid biosynthesis and drives cholesterol production—emerged as a central regulator of PEMF-promoted neuronal differentiation and synaptic maturation (Fig. 6 and 7). PEMF robustly upregulated *FDFT1* expression and its downstream steroid biosynthesis effectors, including *SOLE, DHCR24, MSMO1, EBP and DHCR7.* Pharmacological inhibition of FDFT1’s squalene synthase activity induced time- and dose-dependent deficits in neurite outgrowth and synaptic protein expression, indicating its essential role in physiological neuronal differentiation. Critically, FDFT1 blockade or genetic knockdown abolished PEMF-induced synaptic maturation, demonstrating that FDFT1-mediated cholesterol biosynthesis is indispensable for the pro-maturational effects of PEMF. Additionally, our work showed that PEMF activated the master regulator of cholesterol biosynthesis SREBP-2 and increased the mRNA level of LDLR but not HMGCR (Fig. 7c–e), suggesting that PEMF directly regulates cholesterol biosynthesis and metabolism independent of the mevalonate pathway.

Chronic exposure to 50–60 Hz electromagnetic fields has been shown to elevate total cortical cholesterol levels. This phenomenon may reflect an adaptive neuroprotective mechanism, as magnetic stimulation triggers free radical release and neuroendocrine stress responses akin to those induced by environmental stressors like heat or light [81]. PEMF (50 Hz, 1 mT) was found to upregulate neurotrophic cytokines such as BDNF in bone marrow mesenchymal stem cell-treated spinal cord injuries [84]. BDNF-mediated cholesterol increases occur selectively in membrane raft domains rather than non-raft regions, suggesting PEMF enhances cholesterol levels via BDNF-mediated regulation of lipid raft dynamics [71]. Future studies should elucidate whether PEMF-enhanced neuronal maturation is mediated through cytokine signaling pathways or via cytokine-modulated lipid dynamics.

A key limitation of this study is the unresolved generalizability of our findings—specifically, whether the PEMF parameters effective in Ngn2-induced neurons are applicable to other neuronal subtypes or translatable to in vivo models and clinical settings. Prior studies using ESCs exposed to high-intensity magnetic fields (0.4 T) have reported significant consequences, including redox imbalance, lysosomal dysfunction, and hyperexcitability—all factors that may compromise cellular proliferation and fate determination [85]. High-frequency magnetic stimulation promotes iPSC differentiation into glutamatergic neurons, whereas low-frequency stimulation primarily increases NeuN expression during neuronal maturation [67]. These studies highlight the necessity of tailoring stimulation parameters to specific cell types, as both variables critically influence experimental outcomes. To advance this field, future studies should systematically map dose–response relationships between magnetic stimulation parameters (e.g., intensity, frequency, duration) and specific neuronal differentiation outcomes.

## Conclusions

In summary, this study demonstrates that PEMF stimulation (1 mT, 15 Hz, 3.75 ms pulse duration) enhances the functional maturation of human iPSC-derived cortical neurons, accelerating synaptic development and improving electrophysiological properties such as excitability and ion channel activity. Transcriptomic profiling revealed that PEMF promotes neuronal maturation by activating cholesterol biosynthesis pathways, with *FDFT1* (squalene synthase) identified as the critical regulator mediating this response. Pharmacological inhibition or genetic knockdown of *FDFT1* abolished PEMF-induced synaptic protein expression and neuronal network formation, confirming its indispensable role. These findings establish PEMF as a non-invasive strategy to improve neuronal differentiation and synaptic maturation through cholesterol biosynthesis modulation, offering promising therapeutic potential for neurological disorders and regenerative medicine applications.

## Supplementary Information

Additional file 1

Additional file 2

## Data Availability

All data generated or analyzed during this study are included in this published article and the supplementary information files. The RNA sequencing data was published in the Sequence Read Archive (SRA) and the BioProject accession number is PRJNA1212769. An uncropped western blot of Fig. 2 and Fig.9 was shown in supplementary Fig. 6.

## Abbreviations

iPSCs: Induced pluripotent stem cells
iNs: IPSC-derived neurons
Ngn2: Neurogenin 2
PEMF: Pulsed electromagnetic field
PSD95: Postsynaptic density protein 95
ESCs: Embryonic stem cells
EB: Embryoid body
MSCs: Mesenchymal stem cells
NSCs: Neural stem cells
BDNF: Brain-derived neurotrophic factor
NSPCs: Neural stem progenitor cells
PBS: Phosphate-buffered saline
SDS: Sodium deoxycholate
PMSF: Phenylmethylsulfonylfluoride
PSD: Power spectral density
DEGs: Differentially expressed genes
GO: Gene ontology
KEGG: Kyoto Encyclopedia of Genes and Genomes
PBST: 0.05% Tween-20 in phosphate-buffered saline
HEPES: N-2-hydroxyethylpiperazine-N'-2-ethanesulfonic acid
V_m_: Resting membrane potential
iAP: Induced action potential
SYP: Synaptophysin
SYN1: Synapsin 1
ER: Endoplasmic reticulum
FDFT1: Farnesyl diphosphate farnesyltransferase
APOE: Apolipoprotein E
SLOS: Smith-Lemli-Opitz syndrome
WT: Wild-type
QPCR: Quantitative real-time PCR
siRNA: Small interfering RNA
