# Supplementary material for: Pulsed electromagnetic stimulation promotes neuronal maturation by up-regulating cholesterol biosynthesis
Source: Stem Cell Res Ther. 2025 Jul 26;16:406. doi: 10.1186/s13287-025-04469-1 (PMC12297729; doi:10.1186/s13287-025-04469-1)
Supplement: Supplementary file 1 — Additional file 1 [file 13287_2025_4469_MOESM1_ESM.docx]

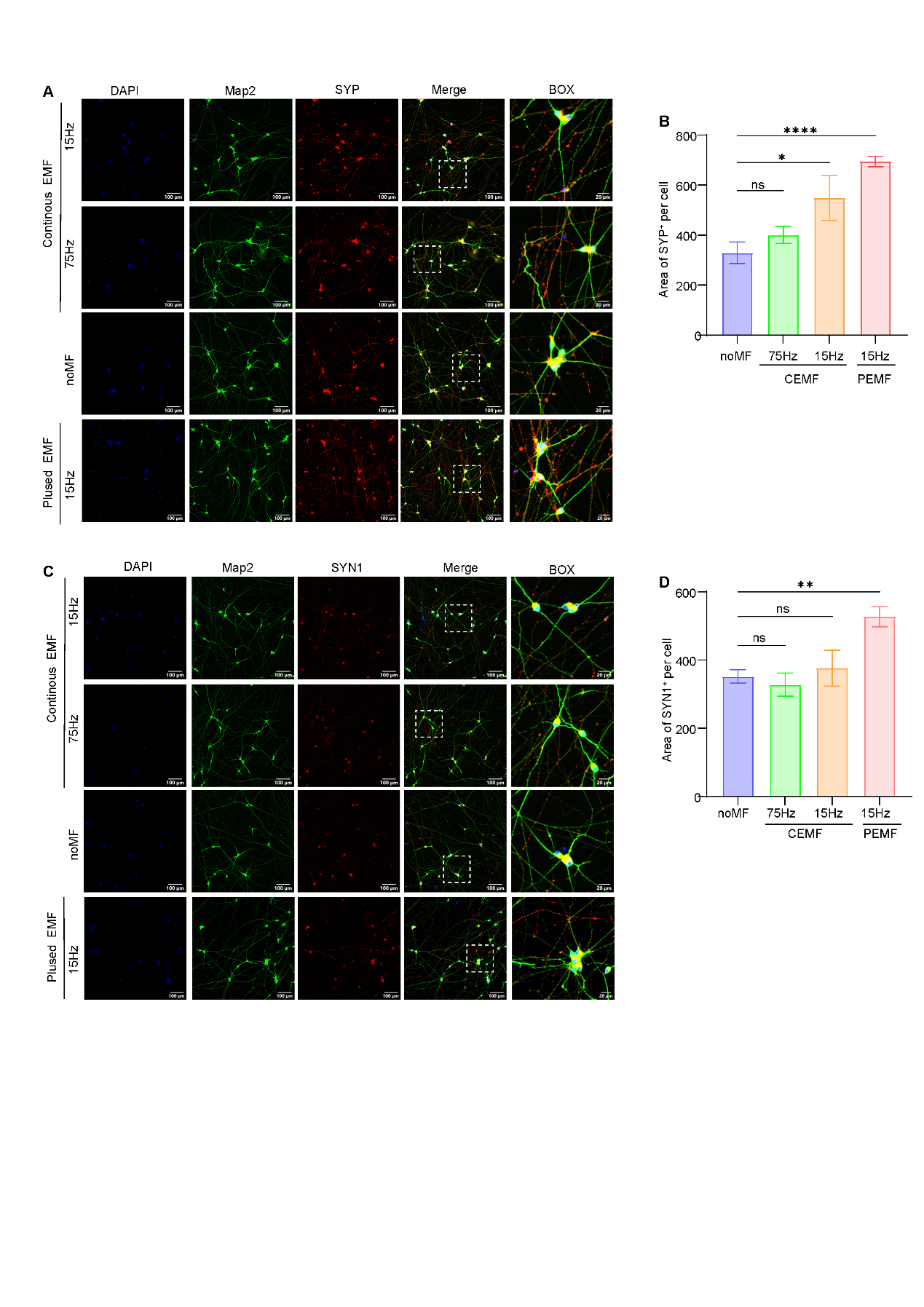


**Supplementary Figure 1. Effect of electromagnetic fields with different parameters on the expression of synaptic proteins in iNs on iN day 11.** (A) Immunocytochemical images produced by a confocal microscope showing synaptophysin expression in iNs stimulated by electromagnetic fields with different parameters. Scale bar: 50 μm. (C) Representative confocal images showing expression of synapsin Ⅰ (SYNⅠ) in iNs from different groups. Scale bar: 10 μm. (B and D) Quantitative analysis of synaptophysin (B) and synapsin Ⅰ (D) in iNs from the different groups on iN day 11. All data are shown as mean ± SEM. Each group has three biological replicates, n=3 in all groups. Treatments were examined by one-way ANOVA and significant differences are denoted as *p < 0.05, **p < 0.01 and ****p < 0.0001.


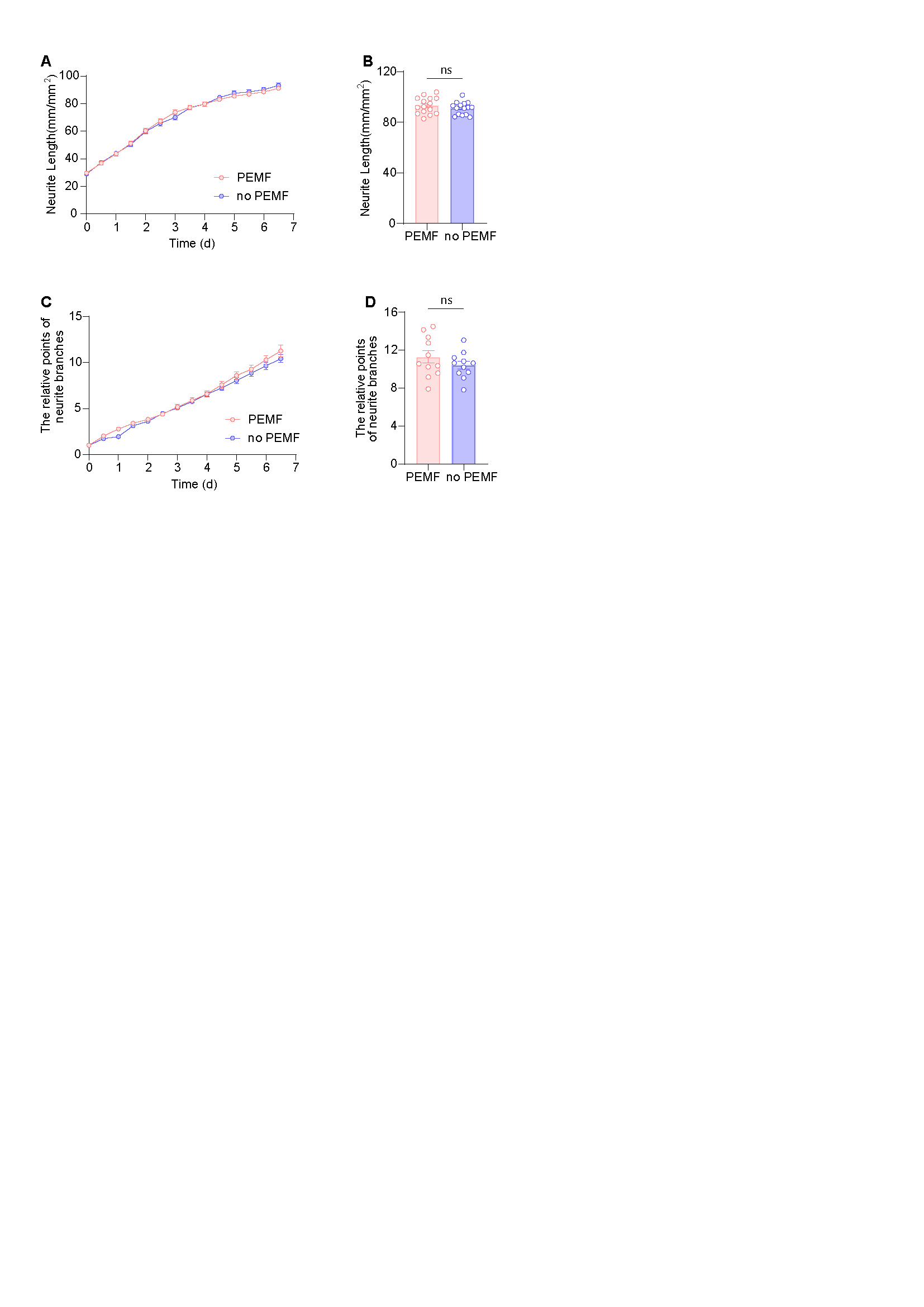


**Supplementary figure 2. Effect of PEMF on the neurite length and branch points of iNs during the process of neuronal differentiation.** (A and B) Time-course analysis of neurite length and branch points by the Incucyte live-cell imaging paradigm starting on iN day 4 indicates PEMF does not affect neurite outgrowth or maturation of branch process. PEMF was administrated in iNs on iN day 5. (C and D) Histogram plots of normalized neurite length and branch points are derived from the last recordings of the traces shown in panel A and B. All data are shown as mean ± SEM and two batches of individual experiment have been performed, n=6 in each group, numbers of sample mean the wells of cultured cells. Treatments were examined by Student’s T-test; ns denotes not significant.

**
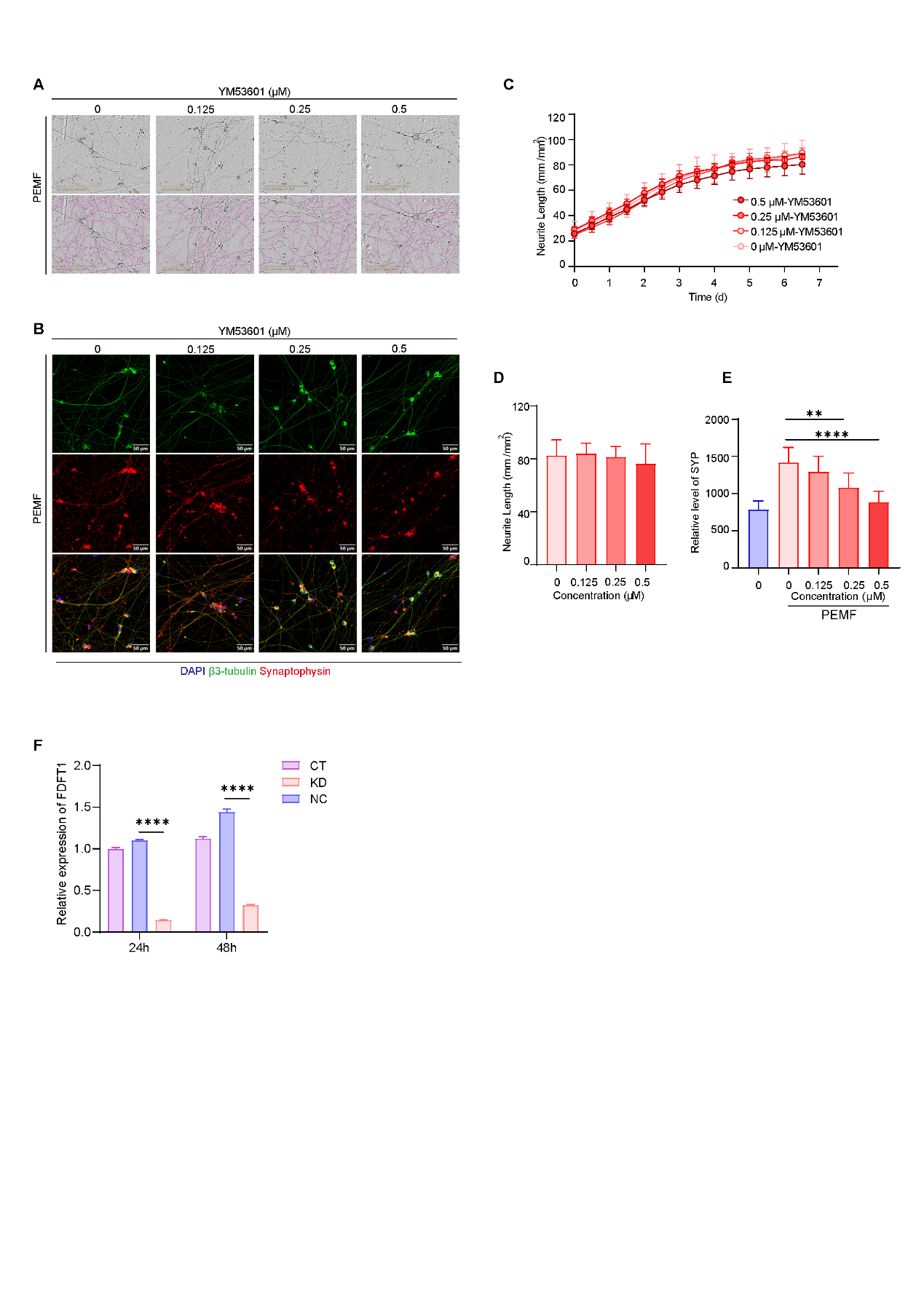
**

**Supplementary Figure 3. YM53601 diminished the promoting effect of PEMF on synaptic maturation of iNs in a concentration-dependent manner.** (A) Representative images and neurite masks of iNs after a 7-day PEMF and YM53601 treatment recorded and analyzed by Incucyte live cell system. The top panel shows phase-contrast images and the bottom panel shows composite images of the identified neurons (purple) and phase-contrast field. Scale bar: 200μm. (B) Immunocytochemical images of synaptophysin (red), β3-tubulin (green) and DAPI (blue) in iNs indicating the expression of synaptophysin of iNs with the exposure of PEMF under the treatment of YM53601 at different concentrations. (C) The outgrowth of neurite analyzed by Incucyte live-cell system with time when iNs were treated by YM53601 at different concentrations and PEMF stimulation. (D) Histogram plots of neurite length derived from the last time point in panel C, n=6 in each group. (E) Quantitative analysis showing the synaptophysin expression of iNs in PEMF groups with YM53601 at different concentrations and CT group, n=4 in each group. (F) Real-time PCR analysis of *FDFT1* level in iNs with different treatments: control (CT), negative control (NC) and *FDFT1-*knocking down (KD), n=3 for each group. All data are shown as mean ± SEM and two batches of individual experiment have been performed. Treatments were examined by one-way ANOVA and significant differences are denoted as **p < 0.01 and ****p < 0.0001.

**
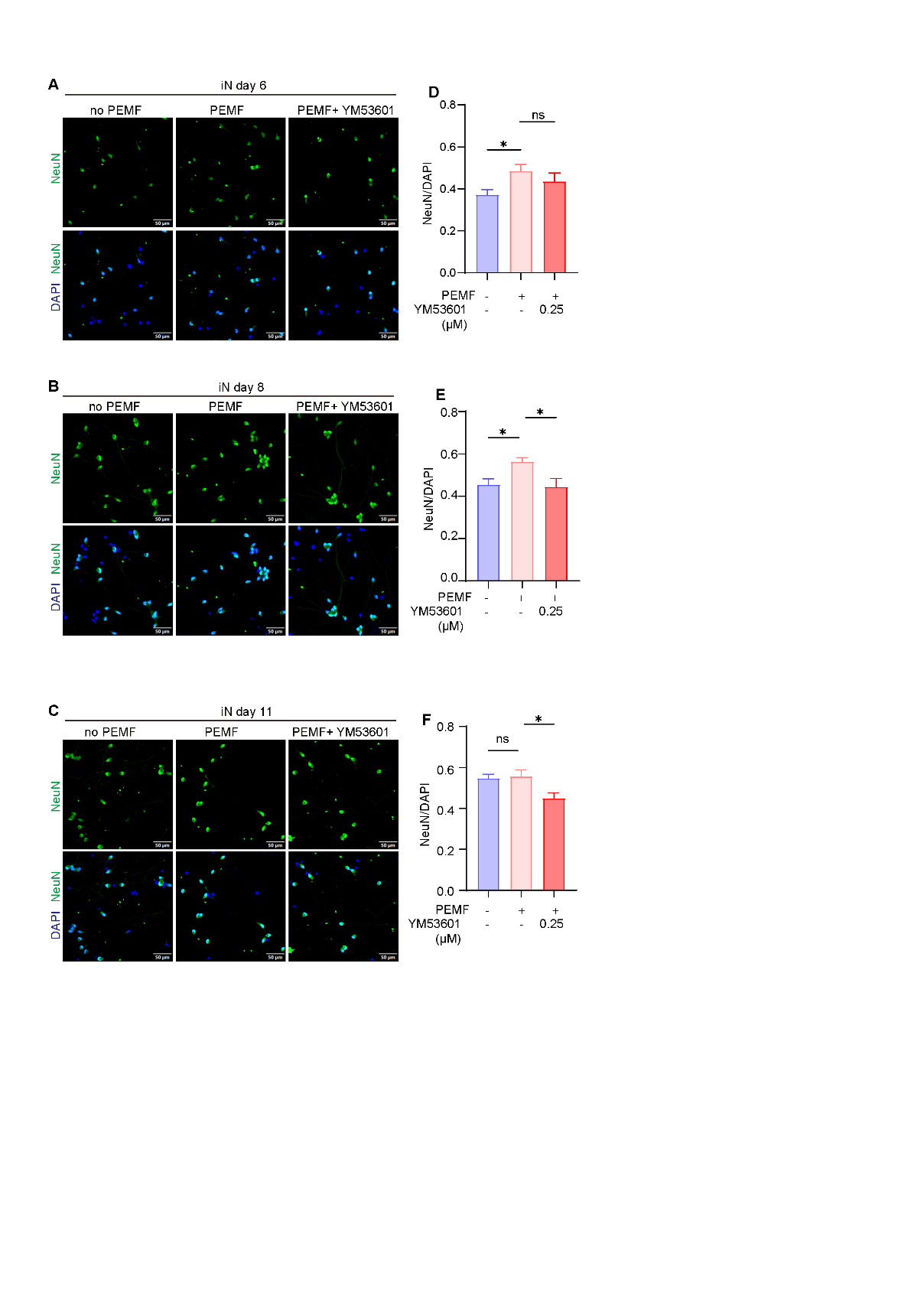
**

**Supplementary Figure 4. Pharmaceutical blockage of FDFT1 diminished the positive effect of PEMF on NeuN expression of iNs at different time points of neuronal differentiation.** (A-C) Representative images of iNs at iN day6 (A), iN day 8 (B) and iN day 11 (C) of neuronal differentiation showing YM53601 attenuated the function of PEMF on increasing the ratio of NeuN to DAPI. ICC was performed by anti-NeuN (green) and DAPI (blue). Scale bar: 50 μm. (D-F) Histogram plots presented statistics on ratio of NeuN to DAPI from three groups at iN day 6 (D), iN day 8 (E) and iN day 11 (F) of neuronal differentiation. All data are shown as mean ± SEM. Each group has three biological replicates, n=3 in each group. Treatments were examined by one-way ANOVA and significant differences are denoted as *p < 0.05; ns denotes not significant.


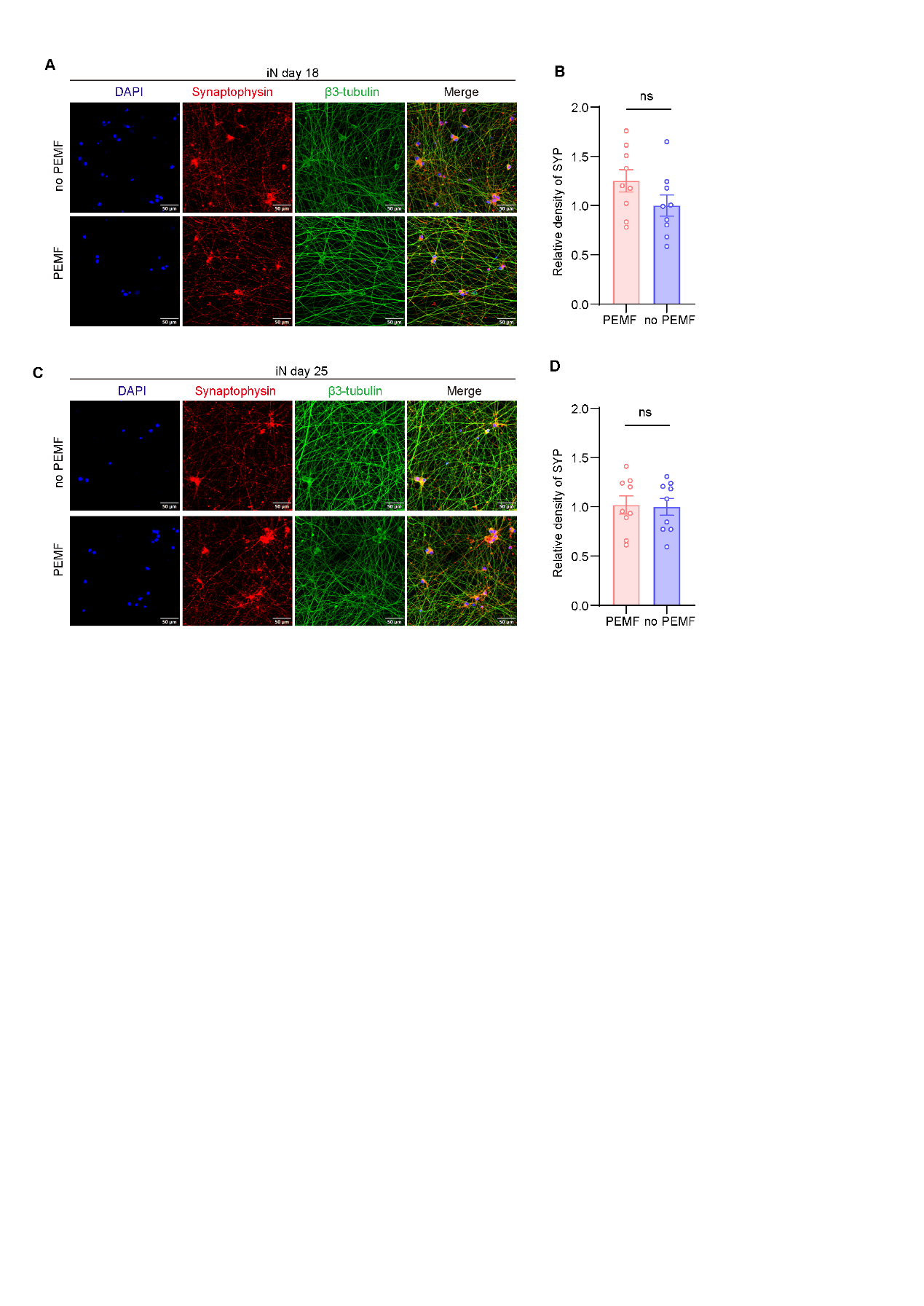


**Supplementary Figure 5. Effect of PEMF on enhanced the expression of synaptophysin diminished over time.** (A) Representative images of iNs treated by PEMF or no PEMF for 7 days and sustained for another 7 days showing the trend of higher expression of SYP in PEMF group but no significant difference. (B) Histogram plots of SYP level in PEMF and no PEMF group from panel A. (C) Representative images of iNs treated by PEMF or no PEMF for 7 days and sustained for another 14 days showing no significant difference of SYP expression in both groups. (D) Histogram plots of SYP level in PEMF and no PEMF group from panel C. All data are shown as mean ± SEM. Each group has three biological replicates, n=3 in each group. Treatments were examined by Student’s T-test; ns denotes not significant.


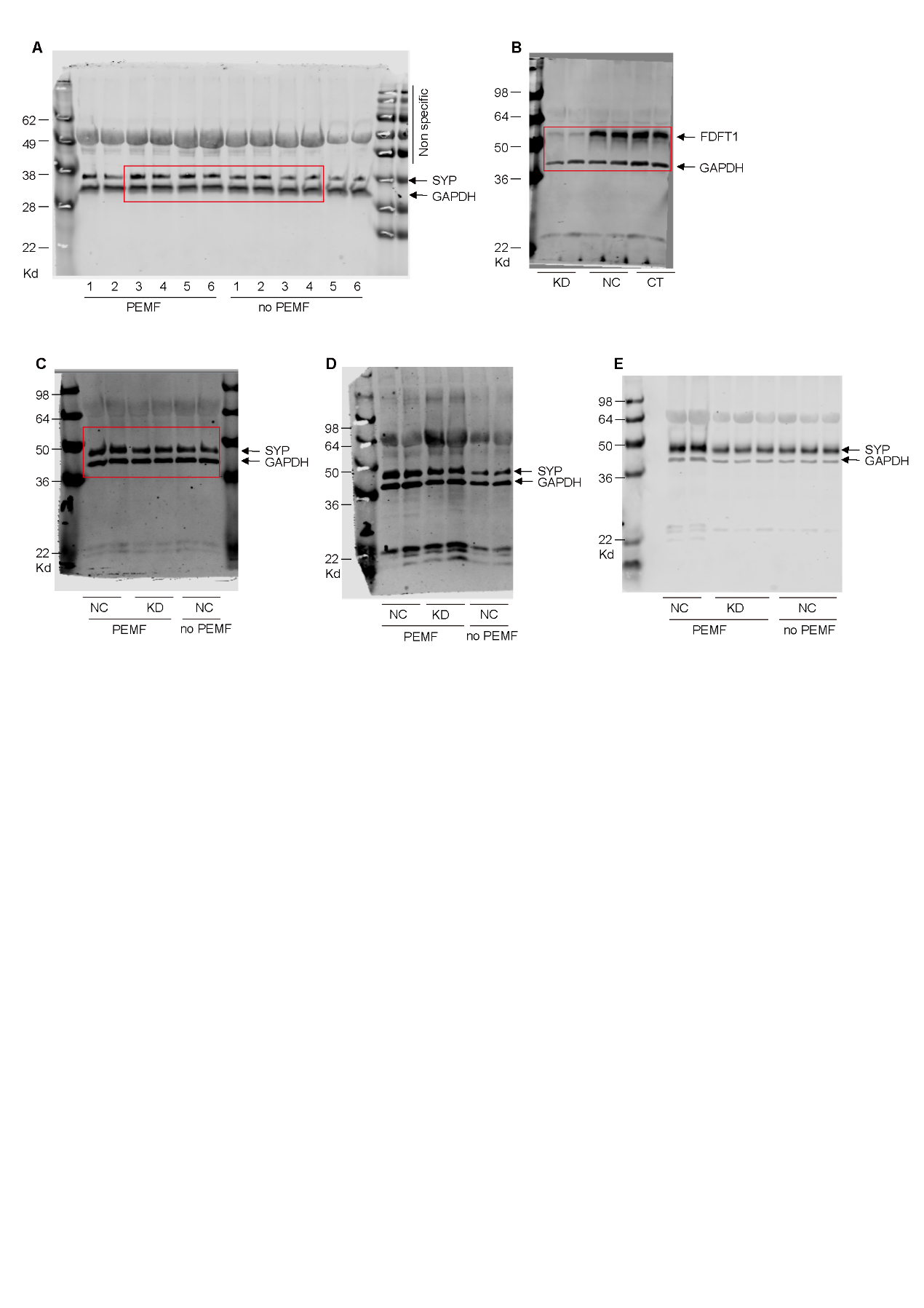


Figure 9H

Figure 9G

Figure 2G

**Supplementary Figure 6. Western blots.** (A) Western blot analysis of synaptophysin in iNs administrated with PEMF for 7 days in Figure 2G. (B) Western blot analysis of FDFT1 in iNs with the treatment of F*DFT1-*siRNA for 7 days in Figure 9G. (C, D and E) Western blot analyzing synaptophysin (SYP) of iNs in response to *FDFT1* knockdown under the PEMF stimulation or not in Figure 9H. GAPDH was used as internal control and three batches of individual experiment have been performed. The red rectangles indicate the cropped immunoblot.


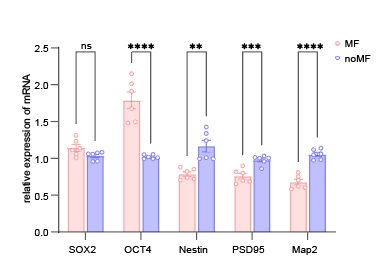


**Supplementary Figure 7. Gene expression of stem cell markers and neuronal markers in iPSCs without the induction of Ngn2 overexpression under the stimulation of PEMF.** Real time qPCR analysis of stem cell markers (*SOX2 and OCT4*), neural development marker (*Nestin*) and neuronal markers (*Map2 and PSD95*) in iPSCs without the induction of Ngn2 treated by PEMF stimulation or not, n=3 in both groups. Results are presented as the mean ± SEM from three independent experiments. Numbers of sample mean the wells of cultured cells. Treatments were examined by student’s t-test and significant differences are denoted as **p < 0. 01, ***p < 0.001 and ****p < 0.0001. ns indicates not significant.
