# Supplementary material for: Pulsed electromagnetic stimulation promotes neuronal maturation by up-regulating cholesterol biosynthesis
Source: Stem Cell Res Ther. 2025 Jul 26;16:406. doi: 10.1186/s13287-025-04469-1 (PMC12297729; doi:10.1186/s13287-025-04469-1)
Supplement: Supplementary file 2 — Additional file 2 [file 13287_2025_4469_MOESM2_ESM.docx]

| **sAP type of activity** | **No PEMF** | | **PEMF** | |
| --- | --- | --- | --- | --- |
|  | **%** | **n** | **%** | **n** |
| Quiet | 97% | 36 | 94% | 32 |
| Attempting | 0% | 0 | 0% | 0 |
| Spontaneous | 3% | 1 | 6% | 2 |

**Supplementary Tables**

**Supplemental Table 1.** Percentage of the types of noninduced activity of DYR0100 human induced pluripotent stem cell (hiPSC)-derived induced neurons (iNs) stimulated by pulsed electromagnetic field for 7 days (PEMF) compared to the control (no PEMF).

| **iAP type of activity** | **No PEMF** | | **PEMF** | |
| --- | --- | --- | --- | --- |
|  | **%** | **n** | **%** | **n** |
| Quiet | 8% | 3 | 0% | 0 |
| Attempting single | 11% | 4 | 3% | 1 |
| Single | 58% | 21 | 52% | 17 |
| Attempting train | 8% | 3 | 27% | 9 |
| Train | 14% | 5 | 18% | 6 |

**Supplemental Table 2.** Percentage of the types of induced activity of DYR0100 human induced pluripotent stem cell (hiPSC)-derived induced neurons (iNs) stimulated by pulsed electromagnetic field for 7 days (PEMF) compared to the control (no PEMF).

|  | **Parameters** | **No PEMF** | | | **PEMF** | | |
| --- | --- | --- | --- | --- | --- | --- | --- |
|  |  | **Mean** | **SEM** | **n** | **Mean** | **SEM** | **n** |
| *Passive* | Vm (mV) | -32.1 | 1.3 | 37 | -38.8*** | 1.3 | 34 |
|  | Cp (pF) | 1.0 | 0.2 | 23 | 0.7 | 0.3 | 5 |
| *Spike analysis* | Threshold (mV) | -36.9 | 1.7 | 29 | -36.0 | 0.9 | 32 |
|  | Overshoot (mV) | 18.0 | 3.2 | 29 | 26.3* | 2.0 | 32 |
|  | Afterhyperpolarization (mV) | -55.6 | 2.3 | 29 | -59.2 | 0.9 | 32 |
|  | Amplitude (mV) | 73.6 | 5.1 | 29 | 85.5* | 2.5 | 32 |
|  | Depolarization rate (V/s) | 42.7 | 5.7 | 29 | 57.3* | 4.2 | 32 |
|  | Repolarization rate (V/s) | -26.9 | 4.7 | 29 | -33.5 | 2.6 | 32 |
|  | Half width (ms) | 4.5 | 0.4 | 29 | 3.2** | 0.2 | 32 |
| *Current* | I Na max (pA/pF) | -1055.4 | 170.1 | 38 | -2967.9** | 586.4 | 33 |
|  | I K max (pA/pF) | 2517.1 | 370.1 | 38 | 4576.6* | 957.6 | 33 |

**Supplemental Table 3.** Functional characteristics of induced action potentials in the DYR0100 human induced pluripotent stem cell (hiPSC)-derived induced neurons (iNs) stimulated by pulsed electromagnetic field for 7 days (PEMF) compared to the control (no PEMF). Abbreviations: Membrane potential (Vm), whole cell capacitance (Cp)., maximal sodium current density (I Na max), maximal potassium current density (I K max). Significantly different values at p<0.05 (*), at p<0.01 (**), at p<0.001 (***).
